# CRISPR-Cas9 library screening combined with an exosome-targeted delivery system addresses tumorigenesis/TMZ resistance in the mesenchymal subtype of glioblastoma

**DOI:** 10.7150/thno.92703

**Published:** 2024-04-29

**Authors:** Jixing Zhao, Xiaoteng Cui, Qi Zhan, Kailiang Zhang, Dongyuan Su, Shixue Yang, Biao Hong, Qixue Wang, Jiasheng Ju, Chunchao Cheng, Chen Li, Chunxiao Wan, Yunfei Wang, Junhu Zhou, Chunsheng Kang

**Affiliations:** 1 Department of Neurosurgery, Tianjin Medical University General Hospital, Laboratory of Neuro-oncology, Tianjin Neurological Institute, Key Laboratory of Post-Neuro Injury Neuro-Repair and Regeneration in Central Nervous System, Ministry of Education and Tianjin City, Tianjin 300052, China.; 2 Department of Neurosurgery, Qilu Hospital, Cheeloo College of Medicine and Institute of Brain and Brain-Inspired Science, Shandong University, Jinan, 250012, China.; 3 Department of Physical Medicine and Rehabilitation, Tianjin Medical University General Hospital, Tianjin, China.

**Keywords:** CRISPR/Cas9, glioblastoma, ERBIN, RASGRP1, VPS28

## Abstract

**Rationale:** The large-scale genomic analysis classifies glioblastoma (GBM) into three major subtypes, including classical (CL), proneural (PN), and mesenchymal (MES) subtypes. Each of these subtypes exhibits a varying degree of sensitivity to the temozolomide (TMZ) treatment, while the prognosis corresponds to the molecular and genetic characteristics of the tumor cell type. Tumors with MES features are predominantly characterized by the NF1 deletion/alteration, leading to sustained activation of the RAS and PI3K-AKT signaling pathways in GBM and tend to acquire drug resistance, resulting in the worst prognosis compared to other subtypes (PN and CL). Here, we used the CRISPR/Cas9 library screening technique to detect TMZ-related gene targets that might play roles in acquiring drug resistance, using overexpressed KRAS-G12C mutant GBM cell lines. The study identified a key therapeutic strategy to address the chemoresistance against the MES subtype of GBM.

**Methods:** The CRISPR-Cas9 library screening was used to discover genes associated with TMZ resistance in the U87-KRAS (U87-MG which is overexpressed KRAS-G12C mutant) cells. The patient-derived GBM primary cell line TBD0220 was used for experimental validations in vivo and in vitro. Chromatin isolation by RNA purification (ChIRP) and chromatin immunoprecipitation (ChIP) assays were used to elucidate the silencing mechanism of tumor suppressor genes in the MES-GBM subtype. The small-molecule inhibitor EPIC-0412 was obtained through high-throughput screening. Transmission electron microscopy (TEM) was used to characterize the exosomes (Exos) secreted by GBM cells after TMZ treatment. Blood-derived Exos-based targeted delivery of siRNA, TMZ, and EPIC-0412 was optimized to tailor personalized therapy in vivo.

**Results:** Using the genome-wide CRISPR-Cas9 library screening, we found that the ERBIN gene could be epigenetically regulated in the U87-KRAS cells. ERBIN overexpression inhibited the RAS signaling and downstream proliferation and invasion effects of GBM tumor cells. EPIC-0412 treatment inhibited tumor proliferation and EMT progression by upregulating the ERBIN expression both in vitro and in vivo. Genome-wide CRISPR-Cas9 screening also identified RASGRP1(Ras guanine nucleotide-releasing protein 1) and VPS28(Vacuolar protein sorting-associated protein 28) genes as synthetically lethal in response to TMZ treatment in the U87-KRAS cells. We found that RASGRP1 activated the RAS-mediated DDR pathway by promoting the RAS-GTP transformation. VPS28 promoted the Exos secretion and decreased intracellular TMZ concentration in GBM cells. The targeted Exos delivery system encapsulating drugs and siRNAs together showed a powerful therapeutic effect against GBM in vivo.

**Conclusions:** We demonstrate a new mechanism by which ERBIN is epigenetically silenced by the RAS signaling in the MES subtype of GBM. Restoration of the ERBIN expression with EPIC-0412 significantly inhibits the RAS signaling downstream. RASGRP1 and VPS28 genes are associated with the promotion of TMZ resistance through RAS-GDP to RAS-GTP transformation and TMZ efflux, as well. A quadruple combination therapy based on a targeted Exos delivery system demonstrated significantly reduced tumor burden in vivo. Therefore, our study provides new insights and therapeutic approaches for regulating tumor progression and TMZ resistance in the MES-GBM subtype.

## Introduction

Glioblastoma multiforme (GBM) is the most aggressive and common primary brain malignancy 1. The incidence rate of GBM ranges from 0.59 to 5 cases per 100,000 individuals and has been rapidly rising in many countries due to certain etiological factors such as aging and the availability of precision diagnosis 2. Currently, the first-line treatment option for newly diagnosed patients is surgical resection combined with radiotherapy plus concomitant adjuvant temozolomide (TMZ). The median survival of GBM patients is around 15 months 3. In these patients, tumor recurrence and drug resistance are the major rate-limiting factors affecting the prognosis of GBM.

Since GBM is a highly heterogeneous carcinoma that gradually invades into the surrounding healthy tissues, complete surgical resection of the tumor is nearly impossible. The combination of radiotherapy and TMZ chemotherapy often fails due to the inherent nature of GBM cells to acquire radio- and TMZ resistance under chronic treatment conditions 4. Histopathological features of gliomas such as necrosis, cellularity, angiogenesis, and mitosis, may not reflect the actual cause of the treatment failure and poor clinical outcomes 5. Multi-dimensional analysis and molecular classification of GBM tumors by The Cancer Genome Atlas Consortium (TCGA) have defined different subtypes of GBM 6, 7. Recent technical advancements in genomic analysis offer precise identification of three major subtypes of GBM, namely the classical (CL), proneural (PN), and mesenchymal (MES) forms 8, 9. Notably, owing to the unique molecular and genetic characteristics of each subtype, the degree of drug sensitivity and treatment responses varies widely from patient to patient. PN and MES subtypes have been extensively studied in the context of treatment outcomes, which reveal that the PN subtype is associated with satisfactory prognosis, while the diagnosis of MES indicates poor survival in GBM patients 10. In addition, during the tumor progression, any GBM subtype can be transformed into another subtype, including the most predominant PN-to-MES transformation (PMT) 11, 12. Interestingly, PMT shares similar cellular characteristics and pathological hallmarks with epithelial-to-mesenchymal transition (EMT) in epithelial-derived solid tumors 13. Studies suggest that radio- and/or chemotherapy can induce the transformation of PN-GBM into the MES subtype, leading to chemo-resistant tumor recurrence and poor prognosis 14.

Comprehensive deep sequencing methods have uncovered how synchronous perturbations of RAS/MAPK and PI3K/AKT signaling and associated cell cycle checkpoint, proliferation, and survival pathways play intricate roles in GBM pathogenesis in adults 6, 7. For example, the MES subtype is characterized by somatic mutations in NF1 and PTEN, modulating the AKT and NF-κB signaling activation 15. NF1 belongs to GTPase-activating proteins, and when it is mutated in GBM, RAS-GTP persists and binds to RAF kinase to initiate the intracellular MEK/ERK phosphorylation cascade, resulting in the activation of the typical intracellular MAPK pathway, promoting cell growth and survival. RAS-GTP can also interact with the p110a catalytic subunit of phosphoinositide3-kinase (PI3K) and enhance its kinase activity to convert PIP2 to PIP3, thereby activating the downstream AKT-p65 signaling pathway to further promote cell proliferation, anti-apoptosis, and chemotherapy resistance 16.

Although KRAS gene mutation is rare in human gliomas, studies have shown that KRAS-G12C can drive the formation of malignant gliomas in mice and KRAS-G12D also plays a role in the progression of astrocytoma to high-grade gliomas 17, 18. KRAS proteins belong to a class of RAS proteins that participate in the RAS-GDP/GTP cycle, and somatic KRAS mutations are present in many different human cancer types. When KRAS is mutated, the conformation of its protein GAP binding site changes, which inhibits GTP hydrolysis mediated by GAP, resulting in constitutive activation of KRAS and continuous downstream signal transmission 19.

CRISPR/Cas9 is a powerful gene editing or manipulation tool offering desired transcriptional and/or functional regulation of the target gene. CRISPR screening is a high-throughput technique in which single-guide RNAs (sgRNAs) are designed, synthesized, and cloned to construct a viral vector-based sgRNA library into a lentiviral library, which is then transduced into cells in a fashion that ensures integration of a single copy of the sgRNA-containing transgene in each cell. The sgRNA complements its target and guides the Cas9 enzyme to a specific DNA location where the Cas9 creates the double-strand break (DSB). Subsequently, repeated rounds of this damage and repair cycle can introduce aberrant mutations in the target gene, resulting in the permanent loss of target gene expression 20. CRISPR screening is highly efficient at identifying genes associated with tumorigenesis, metastasis, immunotherapy response, and drug resistance 21.

Most drugs tested against GBM do not work well in the long run because of their functional incompetency to penetrate the blood-brain barrier (BBB). More than 10 targeted drugs have been approved for clinical trials, but only a few could improve the overall survival in GBM 22. To address this, various vector systems have been designed for the co-delivery of RNA and drug agents specifically to GBM. This approach inevitably brings in certain technical complications, including the system-specific complexity; variations in the packaging of therapeutics across batches, and unknown metabolism pathways that may hinder the clinical translation of the therapeutic agent 23-25. Biosystems-derived exosomes (Exos), which are composed of amphiphilic lipid bilayers surrounding the aqueous nucleus, offer a unique opportunity for co-loading of drugs with different physicochemical properties, which is otherwise a quite challenging scheme 26, 27. In this context, blood-derived Exos comprise a practical delivery system for clinical transformation procedures, especially in individuals with allogeneic blood 28. Interestingly, transferrin receptors (TfR) that express on the surface of blood Exos have been shown to promote BBB penetration without requiring any remarkable structural modifications 29. This approach deserves further exploration to optimize for different drug combinations to address tumor progression and drug resistance in GBM.

Here, due to the lack of primary MES subtype cell lines, we overexpressed the KRAS-G12C mutant to construct GBM cells with sustained RAS signal activation to simulate MES-GBM characterized by RAS signal activation caused by NF1 mutation. We performed the CRISPR-Cas9 library screening with or without TMZ treatment. Through an integrated analysis of the raw data using the MAGeCKFlute algorithm, we discovered a new mechanism by which ERBIN (Erbb2 interacting protein) could be epigenetically silenced by RAS. Further, inhibition of the RAS signaling by EPIC-0412 (EPIC) showed significant restoration of the ERBIN level in GBM cells. RASGRP1 and VPS28 promoted TMZ resistance by facilitating the transition of RAS-GDP to RAS-GTP and increasing the TMZ efflux, respectively. Finally, we showed that an Exos-based quadruple combination therapy could significantly reduce the tumor burden *in vivo*. Our study thus provides new insights and therapeutic approaches to limit the GBM tumor progression and development of TMZ resistance in the MES-GBM subtype.

## Methods

### Cell culture, lentiviruses, and reagents

The patient-derived primary GBM cell line TBD0220 was used for this study, as described previously 30. Human GBM cell line U87-MG was purchased from the American Type Culture Collection (ATCC) and cultured in DMEM medium supplemented with 10% fetal bovine serum (FBS). Cells were cultured in a humidified incubator at 37°C in 5% CO_2_. EPIC-0412 was synthesized by WuXi AppTec Company (WuXi, Jiangsu, China). TMZ was purchased from Sigma. Cells were seeded 24 h before the drug treatment.

For KRAS-G12C overexpression, we used an LV-KRAS-G12C lentivirus (GV218 vector) from GENECHEM (Shanghai, China). All U87-KRAS or TBD0220-KRAS or OE-KRAS descriptions in the whole article represent overexpression of KRAS-G12C mutations in GBM cell lines, which we refer to as U87-KRAS or TBD0220-KRAS or OE-KRAS for short to facilitate description and organization of the Figure. For ERBIN overexpression, we used pLVX-CMV-MCS-3flag-EF1-puro lentivirus from Ibsbio (Shanghai, China). First, the target cells to be transfected were inoculated into the culture dish, and when the cell fusion degree reached 30%, the lentivirus was added to the cell culture medium according to the MOI value recommended in the instructions. After continuing to culture the cells for 72 hours, 2 μg/mL puromycin was added to screen the positive transfected cells for 1-2 weeks. The transfection efficiency was tested by WB, and the cells with stable transfected were used for subsequent experiments (**Figure S1A-B**).

For gene knockdown (KD) experiments, cells were transfected with respective small interfering RNAs (siRNA; Ibsbio, Shanghai, China), as per the previously described protocol 31. First, the cells were prepared and seeded onto a 96-well plate or a 10 cm cell culture dish. Then Lipo3000 was mixed with serum-free medium and labeled as component A for reserve. In next, siRNA was mixed with serum-free medium and labeled as component B for reserve. The prepared component A and B are mixed and labeled as component C and left for 20 minutes. Prepare a serum-free medium to replace the cell medium in cell plates or cell culture dishes. Component C was added to the above cell plates or cell culture dishes and cultured for 4-6 hours to replace the conventional cell medium. All the experiments included targeted siRNAs and non-targeting siRNAs as negative controls. The transfection efficiency was tested by PCR or WB (**Figure S1C-H**). The siRNA sequences used are listed in **Table S1**.

### Genome-Wide CRISPR screening

The U87 and U87-KRAS cells were infected with the GeCKOv2 human lentiviral library (GENECHEM, Shanghai, China) at a multiplicity of infection (MOI) of 0.3 to ensure that most cells received only one stably integrated sgRNA cassette. On day 2, puromycin was added to the media for selecting positively transduced cells. On day 9, the cells were treated with indicated concentrations of TMZ (350 μM). After 14 days of TMZ treatment, cells were harvested, and genomic DNA was isolated for the amplification of sgRNAs-encoded regions, which were then sequenced on an Illumina X platform, and the raw data was analyzed by MAGeCKFlute 1.6.5 as described previously 21.

### RNA isolation and qRT-PCR assay

Total RNA was extracted using TRIzol reagent (Invitrogen) from cells, and then 1µg of RNA was used to synthesize respective cDNA using the PrimeScript RT Reagent kit (Takara). Analysis was performed in triplicates using the SYBR Green reaction mix (Takara) on QuantStudio 3 Real-Time PCR system (Thermo Fisher Scientific). The primer sequences used for the qRT-PCR are listed in **Table S1**.

### RNA sequencing

Total RNA was isolated and amplified to construct the cDNA library, following the manufacturer's instructions, for sequencing on the Illumina HiSeq 4000. Next, sequence data were aligned to the reference genome hg19 using Hisat2 v2.0.5. The expression levels of differentially regulated genes were measured by Fragments Per Kilobase per Million mapped fragments (FPKM).

### Active RAS pull-down assay and Western Blotting (WB)

Levels of RAS-GTP were determined using an active RAS pull-down and detection kit, according to the manufacturer's protocol (Thermo Fisher Scientific, #16117). Western blot (WB) analysis was performed as described previously 32. Antibodies used in this study are shown in **Table S2**.

### Chromatin Immunoprecipitation (ChIP) assay

ChIP assays were performed using the Magna ChIP™ A/G Chromatin Immunoprecipitation Kit (EMD Millipore, #17-10085), as per the manufacturer's instructions. Purified DNA samples were quantified by the qPCR method. The primer sequences used in this assay are listed in **Table S1**.

### Chromatin Isolation by RNA Purification (ChIRP)

Magna ChIRP RNA Interactome kit (EMD Millipore, #17-10494) and Magna ChIRP Human HOTAIR lncRNA Probe Set (EMD Millipore, #03-312) were used, as described elsewhere 32. DNA samples eluted from the pulled-down complex were amplified by PCR. The primer sequences used are listed in **Table S1**. Also, eluted protein samples were analyzed by WB.

### Cell viability, clonogenic, and transwell assays

Approximately 2×10^3^ cells were seeded per well in 96-well plates before drug treatment. The Cell Counting Kit-8 (CCK-8; Dojindo) was used to evaluate the cell viability at indicated time points. For clonogenic assays, about 300 cells were seeded per well in 6-well plates followed by drug treatment and culturing for 12 days. Colonies were then fixed with 4% paraformaldehyde (PFA) and visualized by staining with a crystal violet solution. Matrigel-coated transwell membrane inserts (Millipore Millicell Hanging Cell Culture Insert, 8 µm) were used for the transwell assay. A total of 1×10^5^ cells in a serum-free culture medium were added to each chamber and placed into 12-well plates containing 10% FBS. After treatment and incubation, the invading cells on the inserts were stained with crystal violet.

### In vivo xenograft mouse model

All animal studies were approved by the Institutional Animal Care and Use Committee (IACUC) of Tianjin Medical University. Four-week-old female BALB/c nude mice were used to construct the orthotopic GBM model. TBD0220 cells (1×10^5^ cells in 3μL PBS), transduced with luciferase-expressing lentivirus, were injected into each mouse under the guidance of a stereotactic instrument at coordinates relative to bregma: 2.0 mm posterior, 2.0 mm lateral, 3.0 mm ventral, to establish the model. Mice were then examined by parietal bioluminescence imaging on days 7, 14, and 21 post-tumor transplantation. Kaplan-Meier survival curves were plotted to measure the overall survival of GBM mice, with or without treatment. The log-rank (Mantel-Cox) test was used to analyze the difference in survival between the groups. After sacrificing animals at their experimental endpoints, intracranial tumor tissues were harvested and fixed for immunohistochemical (IHC) analysis.

### Hematoxylin and Eosin (H&E) and IHC analyses

H&E staining and IHC assays were performed as previously described 33. The primary antibodies used in IHC analysis are listed in **Table S2**.

### Confocal Immunofluorescence (IF) microscopy

IF and laser confocal imaging were performed as previously described 34. Antibodies and working dilutions used in this assay are described in **Table S2**.

### Transmission Electron Microscopy (TEM)

Cells were fixed with 2.5% glutaraldehyde, then treated with 1% osmium tetroxide solution, followed by dehydration in graded ethanol and drying with hexamethyldisilazane. After embedding into epoxy resin, samples were sliced into sections with a thickness of 70 nm and stained with 2% uranyl acetate and lead citrate. Finally, slides were sputtered with gold-palladium and imaged by an H7760 microscope.

### Exos isolation and characterization

Previously described protocols were used for Exos isolation and characterization 35. Before extracting Exos, we first quantified the cells of different groups and adjusted the volume of cell culture supernatant collected according to the quantitative results to ensure that the obtained cell culture supernatant was from the same number of cells. At this point, the number of exosomes extracted from these cells reflects the exocrine capacity of the above cells. Next, we extracted protein from the above extracted Exos, dissolved protein precipitation with the same volume of Loading buffer, and loaded the same volume for WB analysis.

### Preparation and Characterization of Exos-drug/siRNA

Exos were isolated and purified from blood samples following the protocol as described elsewhere 36. The total protein content and particle concentration of Exos were measured by the BCA method and nanoparticle tracking analysis (NTA), respectively. To load the Exos with TMZ or EPIC, 100 μg of purified Exos were gently mixed with 100 μg of TMZ or EPIC in 200 μL electroporation buffer in cuvettes, followed by electroporation at 350 V and 150 μF using a Bio-Rad Gene Pulser Xcell Electroporation System. After that, the mixture was incubated at 37°C for 30 min to allow the recovery of the exosome membrane. Then, the buffer was replaced with RNase-free PBS to prepare Exos for loading with siRNAs. Briefly, drug-loaded Exos (Exos-drug) and cholesterol-conjugated siRNAs (chol-siRNAs) were gently shaken at room temperature for 1 h. Exos were then washed with PBS by ultracentrifugation to remove unbounded siRNA. The size distributions of Exos and Exos-Drug/siRNA were analyzed by NTA (NanoSight NS300, Malvern). TEM was used to observe the morphology of Exos and Exos-Drug/siRNA. Exos-specific marker (CD63, CD81, Tsg101, TfR) expressions were then measured by WB.

### *In vivo* BBB penetration and intertumoral distribution assessment

Mice were intravenously (i.v) injected with Cy5.5-labeled Exos or Exos-Drug/siRNA at 14 days post-implantation (dpi). At 1, 2, 4, 6, 12, and 24 h timepoints, the bioluminescence and the fluorescence of Cy5.5-labeled Exos in the brain region were monitored by an *in vivo* imaging system (IVIS) (Spectrum, Caliper, USA) at 675 nm excitation and 720 nm emission wavelengths. For *ex vivo* imaging, mice were sacrificed at 24 h, and their brains were collected, fixed, and processed for IF analysis. The fluorescence intensity was quantitatively evaluated using Living Image 3.1 software.

### Liquid Chromatography- Mass Spectrometry (LC-MS) sample preparation

Animals were transcardially perfused with ice-cold saline. The tumor and contralateral brain tissues were harvested and frozen promptly in liquid nitrogen (N_2_). Tissues were homogenized in a frozen grinding instrument at 4°C and 65 Hz grinding speed for 4 min and stopped for 5 s per minute. Then, 100 µL sample was mixed with 100 µL of 50% methanol solution containing 0.1% formic acid, 50 µL internal standard fluid, and 1 ml of ethyl acetate containing 0.1% formic acid. Then the mixture was centrifuged at 12,000 g for 10 min. The supernatant was collected, dried under N_2,_ and resuspended in 100 µL of 50% methanol solution containing 0.1% formic acid. Finally, 5 µL of the supernatant was injected into the LC-MS system for the concentration analysis of EPIC.

Cells in different groups were counted and then adjusted the volume of the collected cell culture supernatant in the corresponding treatment group according to the number of cells, and then carried out exosome extraction. We strictly ensured that the extracted Exos came from the same number of cells, and then performed LS-MS detection to characterize the level of TMZ changes in the extracted Exos. Cell and Exos pellets were resuspended in methanol and extracted using a cell disruptor for 10 min (power 200 W, pulse for 3 s, with a 2 s interval). After that, the samples were centrifuged at 4°C and 16,000g for 10 min, and 10 μL of the supernatant was injected into the LC-MS system for the concentration analysis of TMZ.

### Tunel assay

Cell slides were washed once with PBS. Then use 4% paraformaldehyde to fix cells for 30 minutes, followed by PBS washing one time. Incubate cells with PBS containing 0.3% Triton X-100 at room temperature for 5 minutes and wash with PBS twice. 50 μL of TUNEL detect solution was added to each sample and incubated for 60 minutes at 37ºC away from light. The cells were washed with PBS 3 times, sealed with an anti-fluorescence quenching sealing solution, and then observed under a fluorescence microscope.

### Statistical analysis

GraphPad Prism 9.0 software was used to conduct all statistical analyses. Student's t-test or analysis of variance (ANOVA) was used for functional analysis. All bioinformatics analyses and visualizations were performed in R 4.2.1. Error bars in the figures represent the mean ± standard deviation (s.d.) from at least three independent (n = 3) experiments. The P < 0.05 was considered statistically significant.

## Results

### Genome-wide CRISPR-Cas9 screening identifies ERBIN in U87-KRAS cells

**Figure 1:**
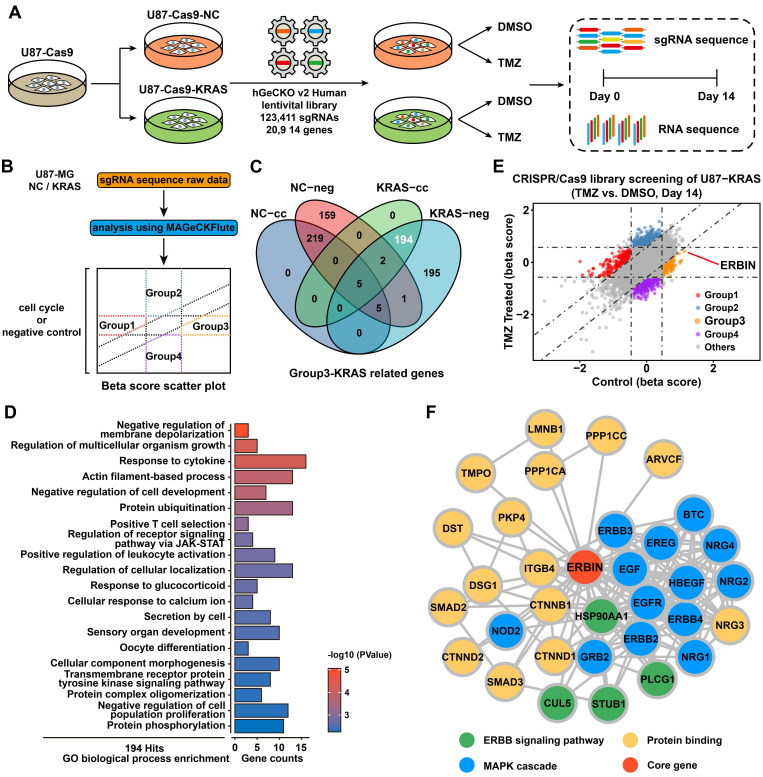
Genome-wide CRISPR-Cas9 library screening identifies ERBIN in the U87-KRAS cells. (A) Schematic representation of the CRISPR-Cas9 library screening and RNA-seq. (B) Analysis of CRISPR screening data. Group 1 represents downstream pathway genes potentially associated with KRAS overexpression. Group 2 represents TMZ treatment-related genes whose deletion leads to TMZ resistance. Group 3 represents KRAS-G12C overexpression-related regulatory genes. Group 4 represents potential TMZ synthetic lethal genes. (C)The Venn diagram of KRAS-related genes. The “cc” represents differential genes obtained by normalization using the “cell cycle” gene set. The “neg” represents differential genes obtained by normalization using the “negative control” gene set. (D) The GO enrichment shows the enrichment of biological process-associated 194 hit genes. (E) The scatter plot of genes obtained by MAGeCKFlute. (F) The Protein-protein interaction (PPI) network diagram of ERBIN.

Due to the lack of primary MES subtype cell lines, we overexpressed the KRAS-G12C mutant in U87-MG to construct GBM cells with sustained RAS signal activation to simulate MES-GBM characterized by RAS signal activation caused by NF1 mutation. By detecting the classic MES marker genes 37, we validated the effectiveness of simulating MES-GBM (**Figure S1I**). Then, we performed CRISPR-Cas9 library screening and RNA-seq in U87-NC and U87-KRAS-G12C (hereafter referred to as U87-KRAS) cells, according to the indicated procedure (**Figure 1A**). The raw data of CRISPR screening were analyzed using MAGeCKFlute. Positively and negatively affected genes were identified and normalized based on the “cell cycle” and “negative control” gene sets and visualized as scatter plots (**Figure 1B**) 21. We identified a total of 194 genes, which were classified as OE-KRAS-associated suppressor genes (**Figure 1C**). Gene Ontology (GO) enrichment analysis of these genes found the transmembrane receptor protein tyrosine kinase signaling pathway and aroused our interest (**Figure 1D**). After screening for genes within the above pathways, we found a tumor suppressor candidate, ERBIN, which ranked in the top position of the Group 3 cluster (**Figure 1E**). In addition, the Ranked plot shows that ERBIN is at the front of the differential gene (**Figure S1J**). Protein-protein interaction (PPI) network analysis of ERBIN indicated its mechanistic linkage with the ERBB and MAPK signaling pathways (**Figure 1F**).

### ERBIN is epigenetically silenced in MES-GBM cells

**Figure 2:**
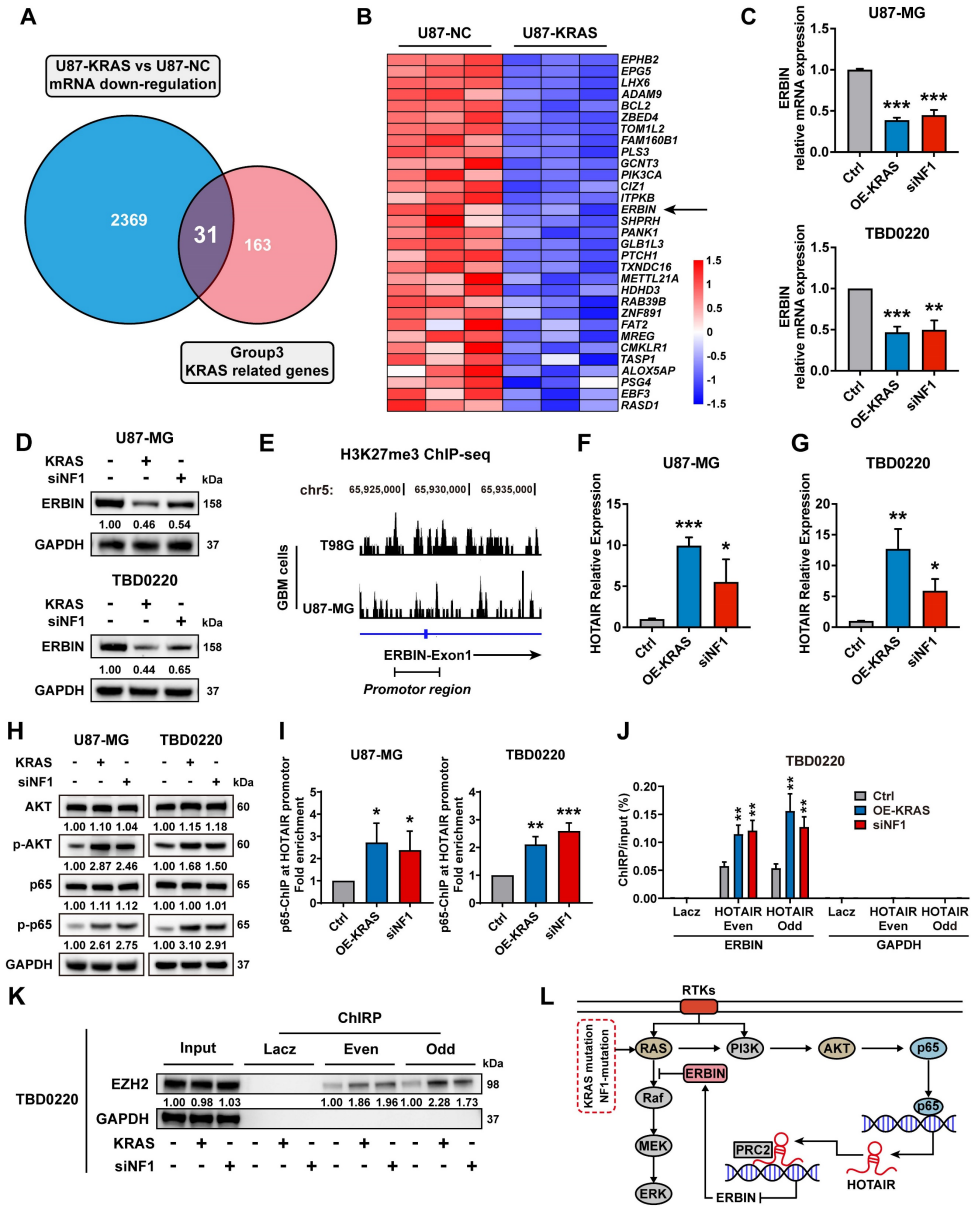
ERBIN is epigenetically silenced in the MES-GBM cells. (A) The Venn diagram of genes from CRISPR screening and mRNA-seq. (B) The heatmap of intersecting genes. (C) ERBIIN mRNA levels in the OE-KRAS, siNF1, and Ctrl GBM cells. (D) ERBIIN protein levels in OE-KRAS, siNF1, and Ctrl GBM cells. (E) The signal peak is located at the promoter region of *ERBIN* in the H3K27me3 ChIP-seq of GBM cells. (F-G) HOTAIR RNA levels in the OE-KRAS, siNF1, and Ctrl GBM cells. (H) Western blot (WB) analysis of AKT, p-AKT, p65, and p-p65 in the OE-KRAS, siNF1, and Ctrl GBM cells. (I) p65 ChIP at the promoter region of HOTAIR in the OE-KRAS, siNF1, and Ctrl GBM cells. (J) HOTAIR ChIRP at the promoter region of *ERBIN* in the OE-KRAS, siNF1, and Ctrl GBM cells. To eliminate non-specific signals, two different pools of probes complementary to the HOTAIR were used (even and odd probe sets). Purified DNA was analyzed by PCR using primers specific to *ERBIN* and *GAPDH* (negative control) genes. (K) ChIRP-WB showed the EZH2 level bound to HOTAIR in the OE-KRAS, siNF1, and Ctrl GBM cells. To eliminate non-specific signals, two different pools of probes complementary to the HOTAIR were used (even and odd probe sets). (L) Diagram of the relationship between the ERBIN and RAS signaling in the MES-GBM subtype. Data are represented as mean ± standard deviation (s.d.); n = 3 independent experiments. (***P < 0.001, **P < 0.01, *P < 0.05).

The RNA-seq data of U87-KRAS cells matched with the CRISPR screening data in **Figure 1** was further analyzed and subsequently intersected with altered genes in **Figure 1C**. In total, 31 genes were detected and visualized in a heatmap. We noticed that ERBIN expression was downregulated in the U87-KRAS cells compared to that in the U87-NC cells (**Figure 2A-B**). We then overexpressed KRAS-G12C mutant (OE-KRAS) or silenced NF1 using siNF1 in the U87 and TBD0220 cells, resulting in the overactivation of RAS and AKT signaling pathways, which mimicked the MES-GBM subtype. We found that OE-KRAS or siNF1 induced depletion of ERBIN at both mRNA and protein levels compared with control group cells (**Figure 2C-D**). We then analyzed ChIP-seq data of GBM cell lines from GEO datasets (GSM3061513 and GSM2573751). The data from GSM3061513 was generated by Vishy Iyer's work, they performed CHIP-seq sequencing of H3K27me3 in GBM cells T98G 38. The data from GSM2573751 was generated by Shuli Xia's work 39, they performed CHIP-seq sequencing of H3K27me3 in GBM cells U87-MG. We found enrichment of H3K27me3 at the *ERBIN* gene promoter region, suggesting that ERBIN might be epigenetically regulated by OE-KRAS or siNF1 in GBM cells (**Figure 2E**). Then we found that OE-KRAS or siNF1 could change the H3K27me3 occupation of the ERBIN promoter by ChIP assay (**Figure 4E**). Previous studies have shown that the PRC2 complex mediates the trimethylation of histone H3 at K27 in a histone methyltransferase EZH2-dependent manner, and this process requires the long non-coding RNA (lncRNA) HOTAIR to play a scaffolding role 40, 41. Hence, we measured expression levels of HOTAIR in OE-KRAS or siNF1-treated cells, which showed that HOTAIR expression was elevated under the above treatment compared to the control group (**Figure 2F-G**). It has been shown that NF-κB triggering can upregulate the HOTAIR expression 42, which we also validated in GBM cells. We found that the AKT-p65 signaling was activated in the OE-KRAS or siNF1-treated cells, with increased p-p65 levels, compared to respective control cells (**Figure 2H**). Subsequent ChIP assay showed increased binding of p65 at the HOTAIR promoter region, demonstrating that HOTAIR was transcriptionally activated by p65 (**Figure 2I**). To explore how HOTAIR might exert an epigenetic effect on the ERBIN promoter region, we conducted the ChIRP assay and found elevated HOTAIR occupancy at the *ERBIN* gene promoter region in OE-KRAS or siNF1 treated groups compared to the control group (**Figure 2J**). Consistently, we also observed enhanced EZH2 binding with HOTAIR at the *ERBIN* gene promoter region (**Figure 2K**). In summary, we found that HOTAIR could increase the level of H3K27me3 by recruiting PRC2 complex to the *ERBIN* promoter region, leading to transcriptional silencing of ERBIN (**Figure 2L**).

### ERBIN overexpression inhibits the RAS signaling and downstream proliferation and invasion effects

**Figure 3:**
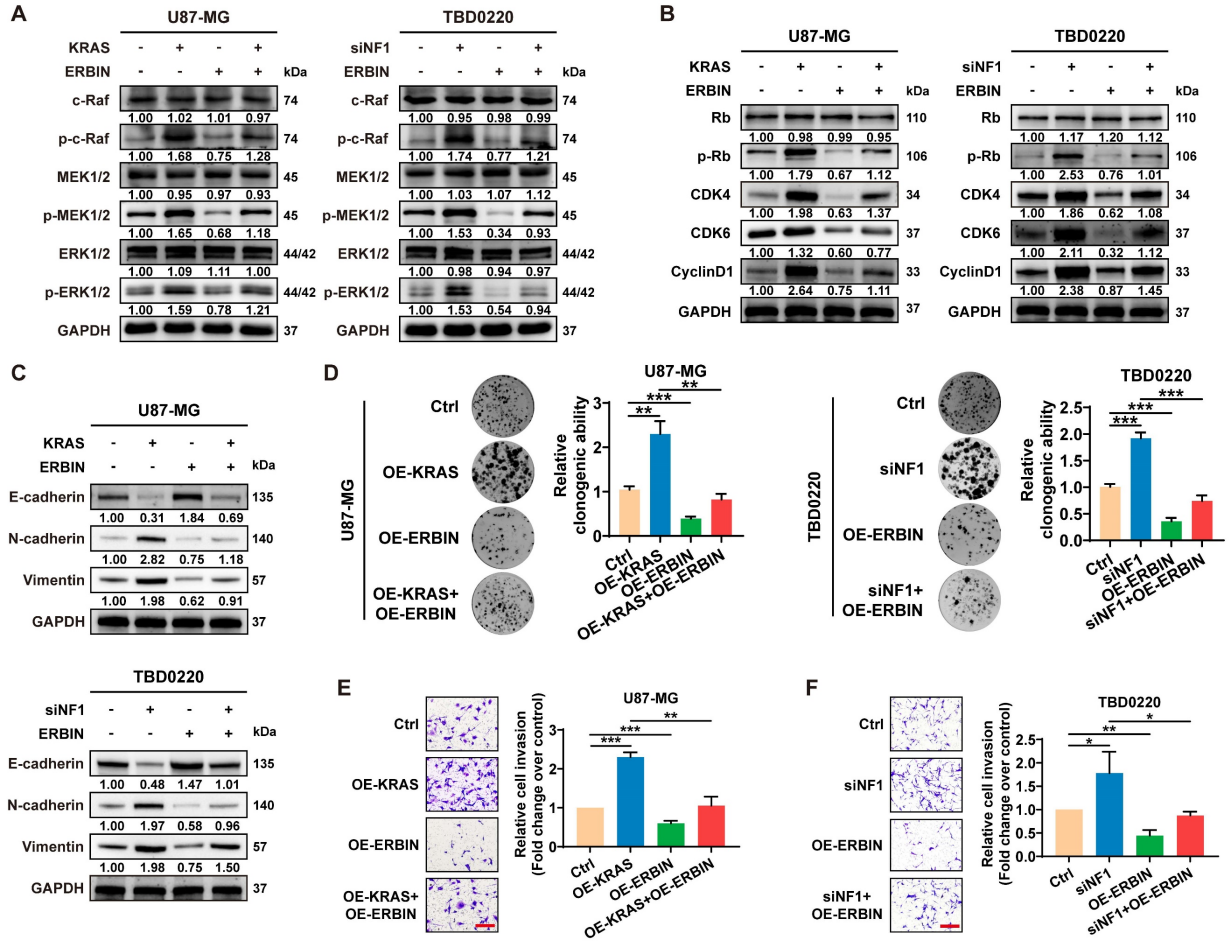
The ERBIN overexpression (OE) inhibited the RAS signaling and downstream proliferation and invasion effects. (A) WB of RAS cascade in the OE-KRAS or siNF1 GBM cells with or without ERBIN OE. (B) WB analysis of p-Rb, CDK4, CDK6, and CyclinD1 in the OE-KRAS or siNF1 GBM cells with or without ERBIN OE. (C) WB analysis of EMT markers in the OE-KRAS or siNF1 GBM cells with or without ERBIN OE. (D) Colony formation assay using the OE-KRAS or siNF1 GBM cells with or without ERBIN OE. (E-F) Transwell assay using the OE-KRAS or siNF1 GBM cells with or without ERBIN OE. Scale bar, 100 µm. Data are represented as mean ± standard deviation (s.d.); n = 3 independent experiments. (***P < 0.001, **P < 0.01, *P < 0.05).

Previous studies have shown that ERBIN expression disrupts the RAS-RAF interaction, thereby inhibiting the downstream ERK signaling 43. Moreover, Erbin was identified as a negative regulator of tumor initiation and progression by suppressing Akt and RAS/RAF signaling in colorectal cancer 44. Here, we observed that overexpressing ERBIN (OE-ERBIN) could attenuate the phosphorylation of RAS signaling downstream c-Raf, MEK, and ERK proteins in U87-MG and TBD0220 cells. In addition, the OE-KRAS or siNF1 treatment resulted in obvious activation of intracellular RAS signals, while OE-ERBIN inhibited this over-activation (**Figure 3A**). Consistently, we observed that OE-ERBIN reduced the protein levels of cell cycle markers p-Rb, CDK4, CDK6, and CyclinD1 in GBM cells. These proteins' levels were upregulated in OE-KRAS or siNF1-treated cells as a downstream effect of the RAS signaling activation and downregulated by OE-ERBIN (**Figure 3B**). Moreover, we found changes in expression levels of EMT indicators, such as upregulation of mesenchymal markers N-cadherin and Vimentin as well as downregulation of epithelial cell marker E-cadherin in OE-KRAS or siNF1 GBM cells, and these changes were reversed by OE-ERBIN (**Figure 3C**). The clonogenic assay also indicated that OE-ERBIN could reduce the proliferation of control group GBM cells, as well as the proliferation induced by OE-KRAS or siNF1 treatment (**Figure 3D**). Moreover, OE-ERBIN inhibited the invasion ability of control group GBM cells. This invasion ability was further promoted by OE-KRAS or siNF1 treatment (**Figure 3E-F**). Overall, our results suggest that ERBIN reduces cell proliferation and EMT processes in the MES-GBM subtype by inhibiting the RAS signaling.

### EPIC-0412 inhibits tumor proliferation and EMT progression by upregulating ERBIN *in vitro* and *in vivo*

**Figure 4:**
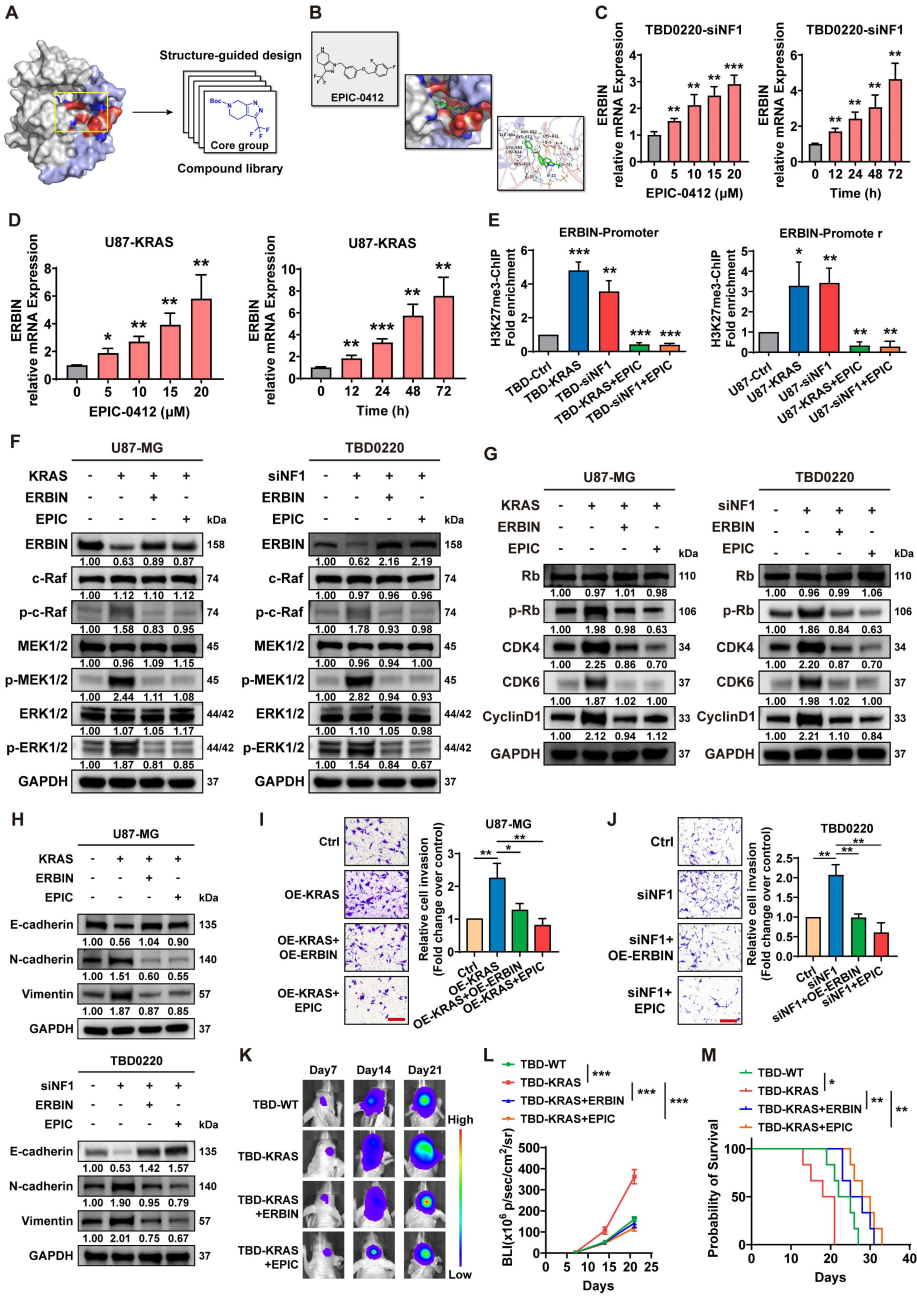
Compound EPIC-0412 inhibits tumor proliferation and EMT progression by upregulating the ERBIN expression both *in vitro* and *in vivo*. (A) Drug screening strategy of EPIC-0412. (B) Molecular formula of EPIC-0412 and its interaction with HOTAIR-EZH2. (C-D) Relative ERBIN mRNA levels in the TBD0220-siNF1 and U87-KRAS cells treated with indicated concentrations of EPIC-0412 for 48 h or 20 µM of EPIC-0412 for indicated time points. (E) ChIP at the *ERBIN* promoter region in the OE-KRAS or siNF1 GBM cells treated with 20 µM of EPIC-0412 for 48 h using an anti-H3K27me3 antibody. WB analysis of (F) ERBIN and RAS cascades, (G) p-Rb, CDK4, CDK6, and CyclinD1, (H) EMT markers in the OE-KRAS or siNF1 GBM cells with or without ERBIN OE and EPIC treatment. (I-J) Transwell assay in the OE-KRAS or siNF1 GBM cells with or without ERBIN OE and EPIC treatment. Scale bar, 100 µm. In F-J, cells were treated with 20 µM of EPIC-0412 for 48 h. (K) Bioluminescence images from the TBD0220-WT, TBD0220-KRAS, TBD0220-OE-KRAS+OE-ERBIN, and TBD0220-OE-KRAS+EPIC groups (15 mg/kg by oral gavage) (n = 6 mice). (L) Quantification of the bioluminescence intensity from all groups. P, two-way ANOVA. (M) Kaplan-Meier survival curve of nude mice. P, Log-rank test. Data are represented as mean ± standard deviation (s.d.); n = 3 independent experiments. (***P < 0.001, **P < 0.01, *P < 0.05).

The above results indicate that ERBIN plays an important role as a tumor suppressor in GBM but is epigenetically silenced by the activated RAS signaling. Next, we sought to unblock the epigenetic silencing of ERBIN mediated by HOTAIR in GBM cells. EPIC is a small-molecule inhibitor that can specifically target the HOTAIR-EZH2 interaction, as revealed by their structure-guided designs (**Figure 4A-B**), as we previously described 32. We found that EPIC treatment significantly increased ERBIN mRNA levels in both dose- and time-dependent manners in MES-GBM cells (**Figure 4C-D**). The ChIP assay further confirmed that EPIC treatment could reverse the OE-KRAS or siNF1-induced increase in the H3K27me3 enrichment at the *ERBIN* promoter region in GBM cells (**Figure 4E**). Next, we found that EPIC inhibited the overactivation of RAS signals in MES-GBM cells by elevating the expression ERBIN (**Figure 4F**). Consistently, we showed that EPIC reduced the level of proteins that were induced in OE-KRAS or siNF1 cells (**Figure 4G**). Moreover, EPIC treatment in OE-KRAS or siNF1 cells restored the expression of E-cadherin in GBM cells. In addition, EPIC administration reduced the OE-KRAS or siNF1-induced upregulation in N-cadherin and Vimentin protein levels in GBM cells (**Figure 4H**). Transwell assay also confirmed that EPIC inhibited the tumor cell invasion activated by OE-KRAS or siNF1 treatment, which had the same effect as OE-ERBIN (**Figure 4I-J**). Next, we constructed an intracranial GBM PDX model in nude mice, which exhibited that tumors in the TBD0220-KRAS group grew at faster rates, conferring a relatively shorter survival time, while the tumors in the TBD0220-KRAS+ERBIN and the TBD0220-KRAS+EPIC groups presented retarded growth. Among these groups, the TBD0220-KRAS+EPIC group had the lowest tumor burden and the longest survival time (**Figure 4K-M**). Moreover, we found that the TBD0220-KRAS group of tumor-bearing mice had low ERBIN expression levels, but high p-MEK and p-ERK expression levels. In the TBD0220-KRAS+ERBIN or TBD0220-KRAS+EPIC group, ERBIN expression was restored, while RAS signaling downstream proteins p-MEK and p-ERK expression levels decreased (**Figure S2**). Taking together, we demonstrate that EPIC can activate *ERBIN* gene expression by perturbing the HOTAITR-EZH2 interaction, which in turn inhibits the activation of RAS signaling and tumor growth *in vitro and in vivo*.

### Genome-wide CRISPR-Cas9 library screening identifies synthetically lethal genes in TMZ-treated U87-KRAS cells

**Figure 5:**
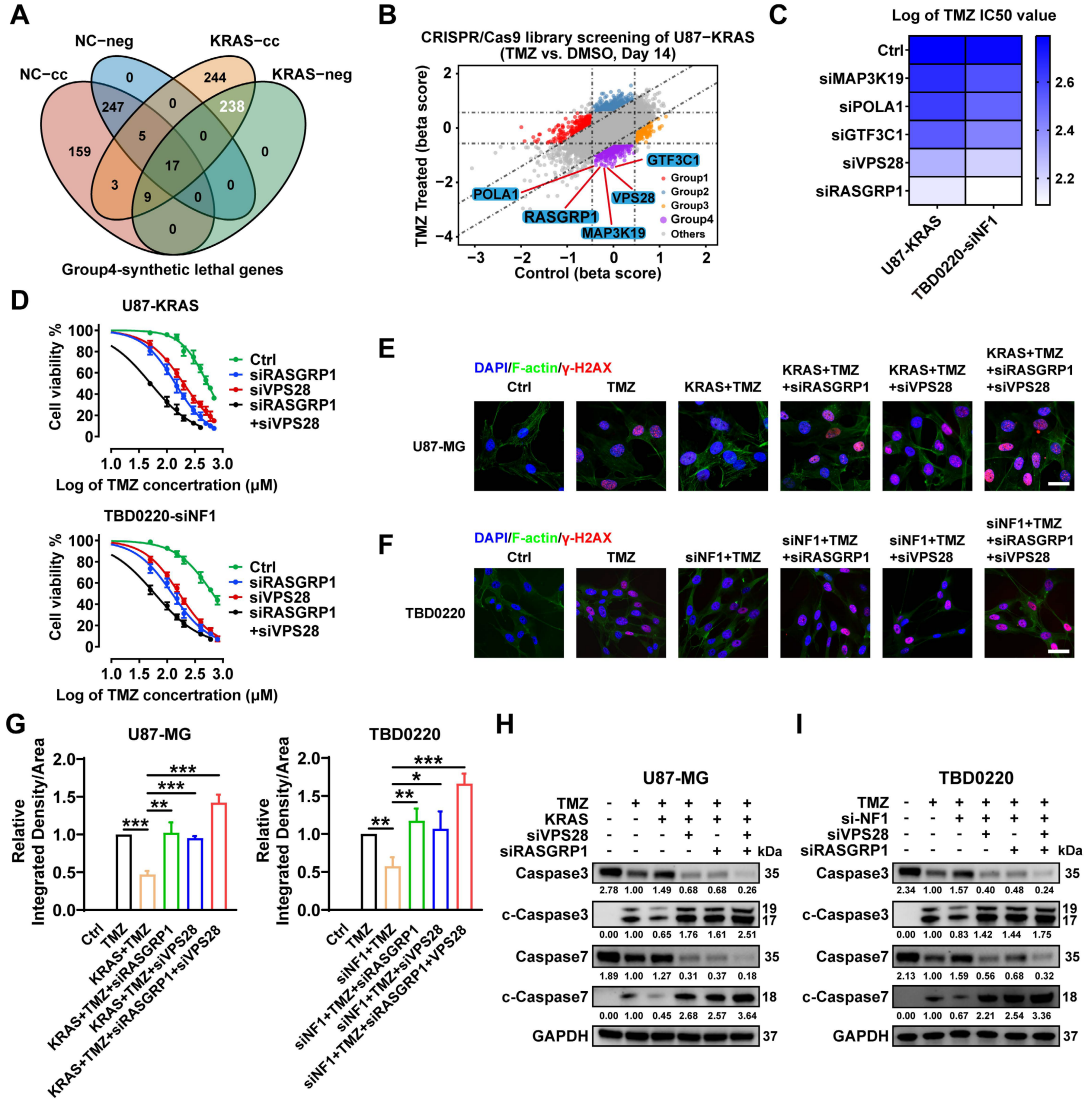
Genome-wide CRISPR-Cas9 library screening identifies *RASGRP1* and *VPS28* genes as synthetically lethal in the U87-KRAS cells treated with TMZ. (A) The Venn diagram of synthetically lethal genes. (B) The scatter plot of genes obtained by MAGeCKFlute shows the top synthetically lethal genes of 238 hits. (C) IC_50_ assay for 48 h treatment of TMZ with downregulation of the top five genes in the OE-KRAS or siNF1 GBM cells, data representing a mean of 3 independent experiments. (D) Cell viability assay for 48 h treatment of TMZ in the OE-KRAS or siNF1 GBM cells with or without RASGRP1/VPS28 KD. (E-G) IF images of γ-H2AX foci in U87 or TBD0220 cells. Scale bar, 30 µm. (H-I) WB analysis of pro- and cleaved-Caspase-3/7. In E-F and H-I, cells were treated with 200 μM of TMZ for 48 h. Data are represented as mean ± standard deviation (s.d.); n = 3 independent experiments. (***P < 0.001, **P < 0.01, *P < 0.05).

In addition to focusing on the genes of the Group 3-KRAS-related cluster, we analyzed the Group 4-TMZ-associated synthetically lethal genes. As shown in the Venn diagram (**Figure 5A**), 238 genes were identified as TMZ synthetically lethal genes. We selected the top five genes as mapped in the scatter plot and carried out subsequent experiments on these genes (**Figure 5B, Figure S3**). *RASGRP1* and *VPS28* genes exhibited the best synergistic lethality upon TMZ treatment in OE-KRAS or siNF1 GBM cells compared to their respective control cells (**Figure 5C**). Furthermore, we found that the IC_50_ of TMZ was much lower when both *RASGRP1* and *VPS28* genes were knocked down in OE-KRAS or siNF1 cells (**Figure 5D**). Besides, OE-KRAS or siNF1 treatment reduced the level of a DNA damage marker γ-H2AX in both U87 and TBD0220 cells with TMZ exposure. However, either *RASGRP1* or *VPS28* KD significantly increased the γ-H2AX level, and this DNA damage effect was more prominent upon knocking down both genes simultaneously in OE-KRAS or siNF1 cells (**Figure 5E-G**). Consistently, we observed weak apoptotic effects of TMZ on U87 or TBD0220 cells treated with OE-KRAS or siNF1. While knocking down either RASGRP1 or VPS28 significantly increased levels of cleaved-caspase 3 and 7, the effect of simultaneously knocking down these two genes on TMZ-induced apoptosis was the most obvious (**Figure 5H-I**). Collectively, these results indicate that RASGRP1 and VPS28 are co-lethal genes of TMZ, knocking down these two genes together can effectively sensitize TMZ in vitro.

### RASGRP1 activates RAS-mediated DNA damage repair by promoting the RAS-GTP transformation

**Figure 6:**
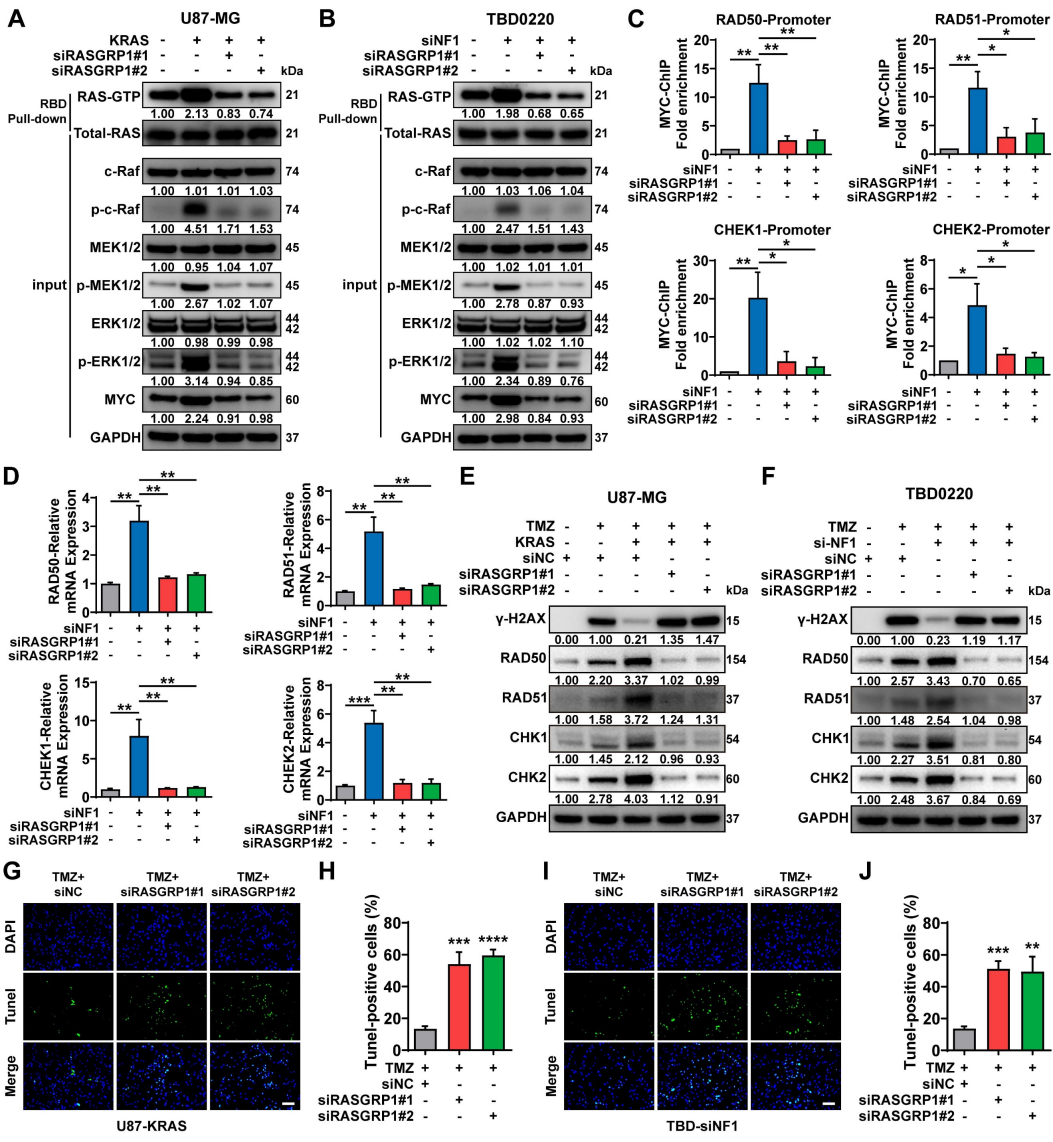
RASGRP1 activates the RAS-mediated DNA damage repair (DDR) by promoting the RAS-GTP transformation. (A-B) The RAS signaling activity and RAS-GTP levels were examined by WB in the U87-KRAS and TBD0220-siNF1 cells treated with siRASGRP1 or siControl. (C) ChIP at the promoter regions of DDR genes in TBD0220-siNF1 cells treated with siRASGRP1 or siControl using anti-MYC antibodies. (D) The mRNA levels of DDR genes in TBD0220-siNF1 or siControl cells treated with siRASGRP1 or siControl. (E-F) WB of γ-H2AX and other DDR proteins after treating OE-KRAS or siNF1 GBM cells with siRASGRP1 with 200 μM of TMZ for 48 h. (G-J) TUNEL analysis of RASGRP1 KD or WT cells with or without TMZ treatment (200 μM, 48 h). Scale bar, 100 µm. Data are represented as mean ± standard deviation (s.d.); n = 3 independent experiments. (****P < 0.0001, ***P < 0.001, **P < 0.01, *P < 0.05).

RASGRP1 catalyzes the release of GDP from small GTPases RAS and RAP to facilitate their transition from an inactive GDP-bound to an active GTP-bound state 45. Here, we observed that either OE-KRAS or siNF1 treatment could activate the RAS signaling and increase the level of the downstream protein MYC in GBM cells. RASGRP1 KD reduced the RAS-GTP transformation, thereby attenuating the downstream RAS signaling cascade, including the MYC expression (**Figure 6A-B**). Previous studies have shown that MYC controls the expression of several DNA double-strand break (DSB) repair-related genes, including RAD50, RAD51, CHEK1, and CHEK2 46, 47.

Here, we found that the enrichment of MYC was significantly increased at the promoter regions of the aforementioned DNA damage repair (DDR) genes due to the activation of RAS signaling in siNF1-treated TBD0220 cells, which could be reverted by RASGRP1 KD (**Figure 6C**). In TBD0220 cells, mRNA levels of RAD50, RAD51, CHEK1 and CHEK2 were increased after siNF1 treatment. However, when RASGRP1 was knocked down at the same time after siNF1, the expression levels of these genes decreased (**Figure 6D**). When OE-KRAS or siNF1-treated U87 or TBD0220 cells were exposed to TMZ, we found that DDR protein levels increased, while γ-H2AX levels decreased. While knocking down RASGRP1 alongside the above treatment weakened the elevated DDR proteins and increased γ-H2AX levels (**Figure 6E-F**). Moreover, siRASGRP1 markedly enhanced the rate of TMZ-induced apoptosis in OE-KRAS or siNF1 GBM cells (**Figure 6G-J**). In summary, our results show that RASGRP1 activates the RAS-mediated DDR signaling by promoting the RAS-GTP transformation, and RASGRP1 KD can restore the TMZ sensitivity in MES-GBM cells.

### VPS28 promotes Exos secretion and decreases intracellular TMZ concentration in GBM cells

**Figure 7:**
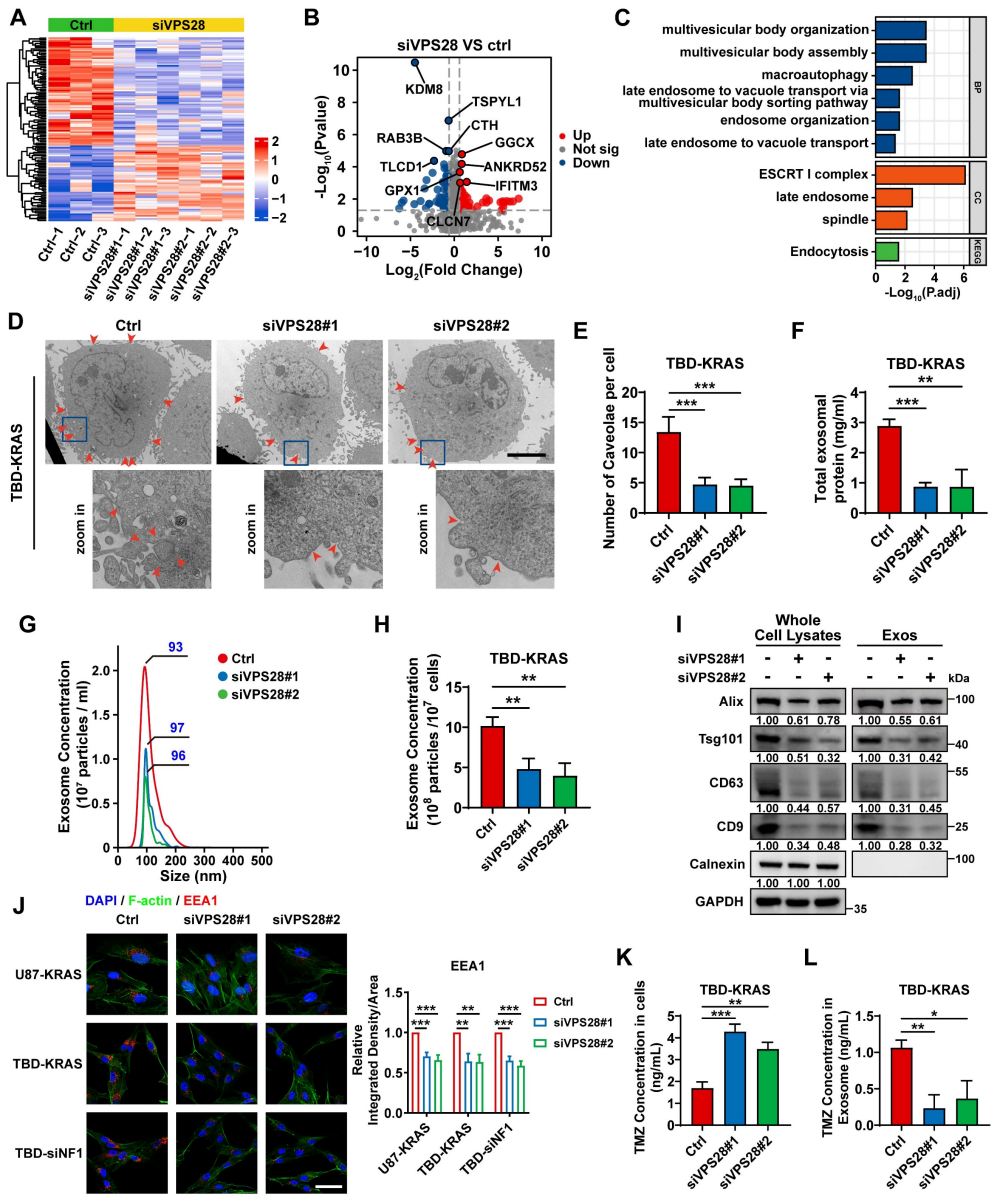
VPS28 promotes the Exos secretion and decreases intracellular TMZ concentration. (A) Heatmap of differentially expressed proteins in the TBD0220-siControl or TBD0220-siVPS28 cells. (B) Volcano plots showing proteins detected by DIA quantitative proteomics in the TBD0220-siControl or TBD0220-siVPS28 cells. (C) Enrichment analysis of differentially expressed proteins. (D-E) Analysis of TEM images and a number of caveolae in the TBD0220-KRAS cells, n = 10. The red arrows indicate caveolae. Scale bars, 5 µm. (F) Total protein levels of Exos secreted by an equal number of TBD0220-KRAS cells with siVPS28 or siControl. (G-H) Quantification and traces of Nanosight analysis for Exos derived from an equal number of TBD0220-KRAS cells with siVPS28 or siControl. (I) WB analysis of Exos markers in the whole cell lysates and Exos from an equal number of TBD0220-KRAS cells. (J) IF images of EEA1 in the U87-KRAS or TBD0220 cells with OE-KRAS or siNF1. Scale bar, 40 µm. (K) Quantification of concentrations of TMZ in the TMZ (200 µM,48 h) treated TBD0220-KRAS cells with siVPS28 or siControl and in Exos from equally treated cells with siVPS28 or siControl. Data are represented as mean ± standard deviation (s.d.); n = 3 independent experiments. (***P < 0.001, **P < 0.01, *P < 0.05).

To unravel the underlying mechanism of the synergic lethal effects of Vacuolar Protein Sorting 28 (VPS28) KD and TMZ on GBM cells, we performed Data-Independent Acquisition (DIA) quantitative proteomic detection in TBD0220-siControl or TBD0220-siVPS28 cells (**Figure 7A-B**). Enrichment analysis of differentially regulated proteins showed that multivesicular body (MVB) organization and assembly, macroautophagy, endosome organization, and endocytosis processes were altered upon VPS28 KD (**Figure 7C**). We hypothesized that VPS28 might play an important role in endosome formation and construction of MVB in exocrine processes. Hence, we performed the TEM assay in TBD0220-KRAS cell line, which revealed that siVPS28 significantly reduced the number of caveolae in TBD0220-KRAS cells compared to that in siControl treated cells (**Figure 7D-E**). Notably, the total protein content in Exos from siVPS28-treated TBD0220-KRAS cells was significantly reduced than that in siControl cells (**Figure 7F**). Nanosight analysis of ultracentrifuged Exos pellets also showed a significant decrease in the number of Exos in siVPS28-treated TBD0220-KRAS cells, compared with the siControl-treated cells (**Figure 7G-H**). As Exos were centrifuged from an equal number of cells, the expression of the exosome surface markers reflected the population of Exos. The siVPS28 treatment significantly decreased the quantity of Alix, Tsg101, CD63, and CD9 markers both in whole cell lysates (WCL) and ultracentrifuged pellets containing Exos. Calnexin was abundant in the WCL but could not be detected in the Exos fraction, confirming the purity of isolated Exos (**Figure 7I**). These results suggest that siVPS28 might have impaired the organization of Exos, resulting in reduced secretion. Next, we measured the level of EEA1, an early endosome marker. We found that VPS28 KD led to a significant reduction in the EEA1 level (**Figure 7J**), indicating an inhibition in the early endosome formation and subsequent Exos secretion than that in siControl treated cells. Our previous studies have shown that GBM cells can excrete TMZ through the exosome secretion pathway, leading to TMZ resistance 35, 48. Here, we observed that VPS28 KD in TBD0220-KRAS cells significantly increased the intracellular concentration of TMZ, while decreasing that in the Exos fraction (**Figure 7K-L**). In summary, our findings suggest that VPS28 may promote Exos synthesis and extracellular secretion by mediating the early endosome formation, which eventually leads to TMZ efflux in the MES-GBM cells. Therefore, VPS28 KD can restore the intracellular therapeutic concentration of TMZ by inhibiting its efflux, thereby enhancing the TMZ sensitivity to GBM cells.

### Construction of the targeted Exos delivery system encapsulating drugs and siRNAs

**Figure 8:**
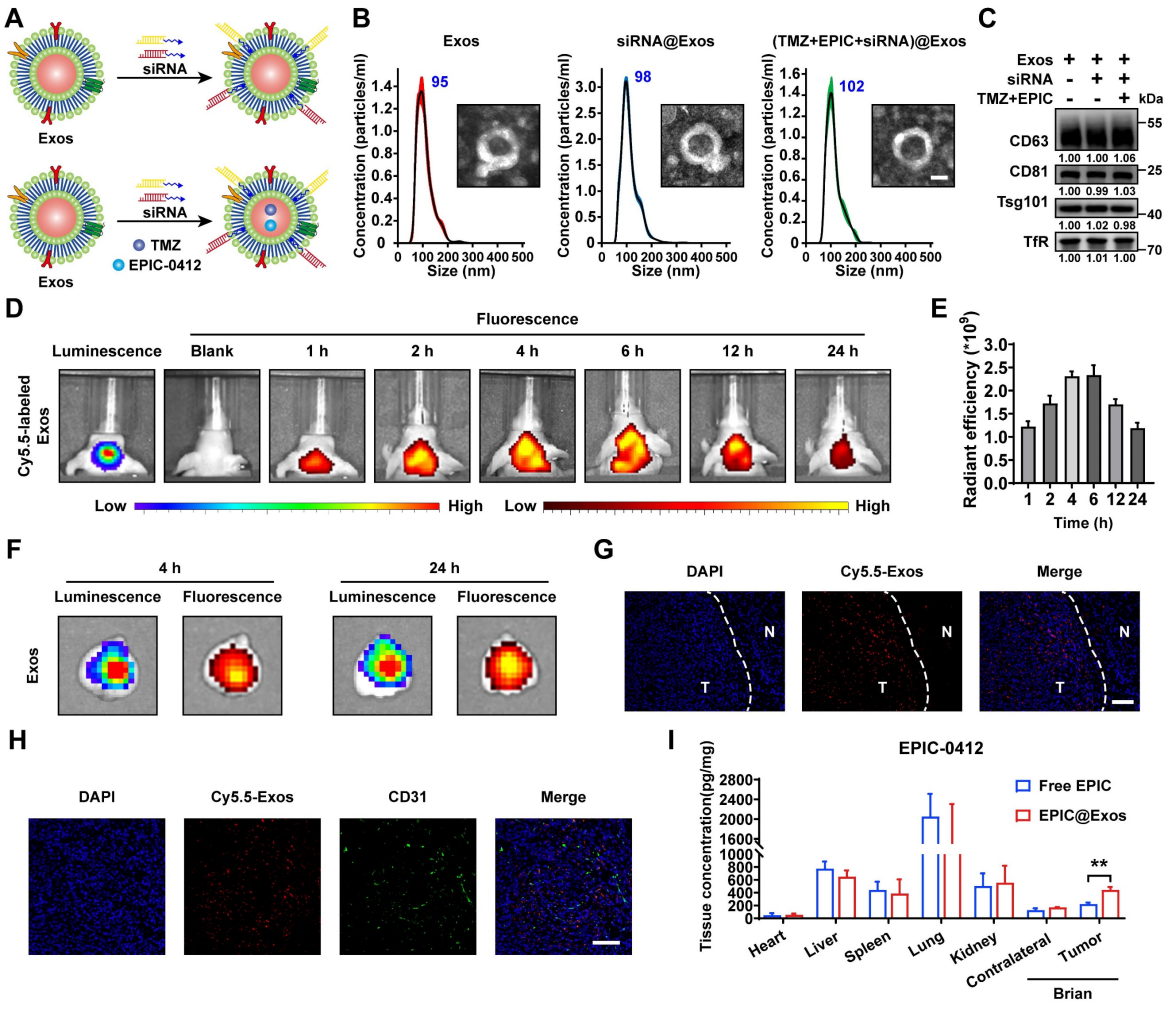
Construction of the targeted Exos delivery system encapsulating drugs and siRNAs. (A) Preparation process of the Exos-based delivery system. (B) Representative TEM images and size distributions of Exos and Exos-Drug/siRNA. Scale bars, 50 nm. (C) WB analysis of CD63, CD81, Tsg101, and TfR expressions in Exos and Exos-Drug/siRNA groups. (D-E) Real-time fluorescence tracking and quantitation of Cy5.5-Exos in the brain regions of TBD0220-Luc-bearing mice (n = 6). (F) Representative *ex vivo* bioluminescence and fluorescence images of brains collected at 4- or 24-h post-injection. (G) Representative confocal images of the brain tissues (T denotes tumor and N denotes normal tissue). Scale bars, 100 μm. (H) Representative confocal images of the GBM tissues. Scale bars, 100 μm. (I) EPIC-0412 concentrations in the major organs of GBM-bearing mice after intravenous injection of free drug or Exos-drug/siRNA at 12 h post-injection. Data are represented as mean ± standard deviation (s.d.); n = 3 independent experiments. (**P < 0.01).

In our previous study, we developed a blood Exos-based delivery system that could co-load sicPLA2(siRNA targeting the cPLA2 gene) and metformin to inhibit mitochondrial energy metabolism in GBM cells, both *in vitro* and *in vivo* 36. Here, we also built a similar delivery system capable of co-loading TMZ and EPIC as well as siRNA targeting RASGRP1 and VPS28 (**Figure 8A**). Both empty Exos and Exos-drug/siRNA exhibited typical "saucer-like" nanoforms. Moreover, the NTA assay showed that after loading therapeutic drugs and siRNAs, the peak particle size of Exos was only slightly increased by 7 nm, without any other significant changes (**Figure 8B**).

WB analysis showed no significant change in the protein levels of typical Exos markers, including TfR, in the Exos, Exos-siRNA, and Exos-drug/siRNA groups (**Figure 8C**). To further evaluate the ability of Exos to cross the BBB *in vivo*, we constructed an orthotopic TBD0220-Luc GBM mouse model. Cy5.5-labeled Exos were injected through the tail vein, and the Exos signal was monitored using an IVIS. We found an increase in fluorescence signals in the brain at 1 h post-injection, and that kept gradually increasing over time, reaching a plateau at 6 h post-injection that lasted until 24 h (**Figure 8D-E**). Ex-vivo*,* fluorescence images displayed fluorescence signals of Exos in the brain and that colocalized with the bioluminescence signal of GBM at 4 h and 24 h timepoints, indicating an effective BBB penetration (**Figure 8F**). IF images of brain tissues further showed that Cy5.5 signals were preferentially accumulated in the tumor region than in the normal brain regions (**Figure 8G**). In addition, IF analysis of the tumor region revealed that most Exos were efficiently exuded from tumor blood vessels and penetrated the GBM tissues, further demonstrating the efficiency of blood-derived Exos in penetrating BBB and specifically accumulating in GBM cells (**Figure 8H**). In TBD0220-grafted mice, injection of Exos or Exos-drug/siRNA exhibited a significant accumulation of EPIC in the brain. At 12 h post-injection, there was a significant accumulation of Exos-delivered EPIC in the brain tumor area compared with that in the contralateral region (**Figure 8I**). Taken together, we successfully show the construction of a targeted delivery system using blood-derived Exos that can co-load therapeutic drugs and siRNAs and are capable of targeted delivery of therapeutics to GBM tumors by efficiently penetrating BBB *in vivo*.

### The targeted Exos delivery system shows a powerful therapeutic effect against GBM *in vivo*

**Figure 9:**
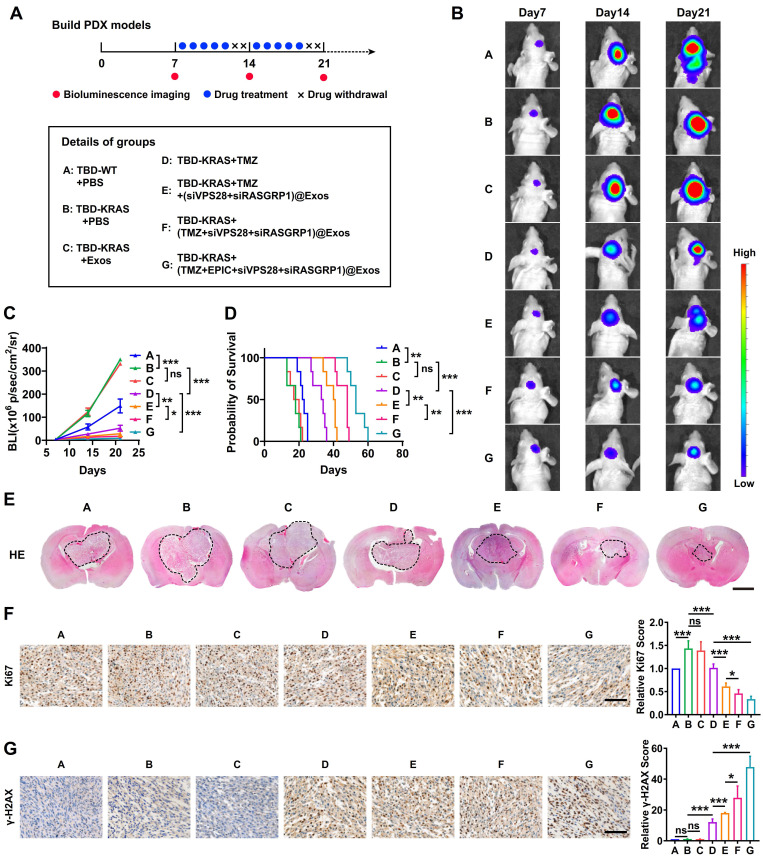
The targeted delivery system shows a powerful GBM therapeutic effect *in vivo*. (A) The timeline and details of the experimental and control animal groups (n = 6). Tumor-bearing nude mice were treated with vehicle, TMZ (5 mg/kg), or Exos-drug/siRNA. (B) Bioluminescence images from representative mice of all the groups. (C) Quantification of bioluminescence intensity from all the groups. P, two-way ANOVA. (D) Kaplan-Meier survival curve of nude mice. P, Log-rank test. (E) Representative images of H&E staining from all the groups of mice. Scale bars, 2 mm. (F-G) IHC images of tumor tissues showing Ki-67 and γ-H2AX expressions. Scale bars, 100 µm. Data are represented as the mean ± s.d.; n = 6 mice. (***P < 0.001, **P < 0.01, *P < 0.05, ns = not significant).

To further explore whether Exos-drug/siRNA could exhibit anti-tumor efficacy *in vivo*, we constructed the PDX-GBM model using TBD0220 cells (**Figure 9A**). Bioluminescence imaging revealed that the group B (TBD0220-KRAS group) had a greater tumor burden and less survival time than the group A (TBD0220-WT group), indicating a strong tumorigenicity of the MES-GBM subtype (**Figure 9B-D**). We then explored the efficacy of both the drug and delivery platform in this MES-GBM PDX model. For group C (native-Exos group), we observed a similar tumor growth and survival time to group B. Compared with group D (single TMZ treatment group), group E (TMZ+Exos-siRNA group) presented a smaller tumor burden and longer survival, while group F (Exos-TMZ/siRNA group) showed significantly enhanced therapeutic effect and improved survival across all the groups. Group G (Exos group containing two drugs and two siRNAs) had the best therapeutic effect and conferred the longest survival time (**Figure 9B-D**). Moreover, H&E staining of brain sections also showed a consistent trend; group G had the smallest tumor burden (**Figure 9E**). IHC analysis also indicated that the Ki67 expression was significantly reduced in group G. We also observed that group D could induce a low level of γ-H2AX foci formation, whereas group E had an increased γ-H2AX level. Group G had the highest level of γ-H2AX (**Figure 9F-G**). Compared with group B, we found that less RASGRP1, VPS28, and RAS downstream proteins p-MEK1/2 and p-ERK1/2 were expressed in mice tumor sections of group G. Moreover, the group G had high levels of ERBIN expression (**Figure S4A**). In addition, we characterized cleaved-Caspased3, a marker of apoptosis. IF analysis showed that group G produced more apoptosis effect than group B (**Figure S4B**). These results together highlight that the combination of siRASGRP1 and siVPS28 with TMZ can enhance the therapeutic effect of TMZ, and the targeted delivery system further enhances the strength of this therapeutic regimen against GBM.

## Discussion

GBM is the first cancer type to be systematically studied by TCGA. The initial work reported relevant biological alterations in three core signaling pathways, namely p53, Rb, and receptor tyrosine kinase (RTK)/Ras/phosphoinositide 3-kinase (PI3K) signaling 6. Subsequent high-throughput analyses and molecular classifications of GBM subtypes by TCGA revealed that the relationship between GBM expression subtypes and genomic abnormalities, treatment response, or tumor microenvironment could differ significantly, resulting in the cellular heterogeneity and plasticity in GBM cells 7, 8, 10. Recently, three clusters CL, PN, and MES have been identified and becoming the widely accepted subtypes of GBM. Patients with the MES subtype have lower survival rates compared to other subtypes, both in primary and recurrent tumors. However, each subtype exhibits a considerable level of dynamic features in GBM patients 49, 50. The PN and CL subtypes often phenotypically switch to the MES upon recurrence, and chronic anti-neoplastic treatments can also alter mesenchymal genetic signatures, suggesting that the transition to MES or EMT-like in GBM may be associated with tumor progression and treatment resistance 37, 51. The most common genetic alteration of the MES subtype is the copy number loss and/or mutation of NF1, resulting in the activation of the RAS/MAPK cascade. Marques et al. have reported that the NF1 loss increases the RAS/MAPK activity and modulates the expression of FOSL1 to maintain the GBM stemness, MES features, and plasticity 52. RAS accepts the transmission of RTK signals from the cell membrane and passes them downward to activate the MAPK signaling pathway. At the same time, RAS also has crosstalk with PI3K, which puts the RAS signaling at the crossroads of MES-GBM.

In this study, we overexpressed the KRAS-G12C mutant to construct GBM cells with sustained RAS signal activation to simulate MES-GBM characterized by RAS signal activation caused by NF1 mutation. We detected hub genes of the RAS signaling and TMZ synthetic lethality genes by the CRISPR-Cas9 screening. ERBIN is described as a suppressor of the RAS-RAF interaction and inhibits the downstream ERK signaling 43. In this study, we found that ERBIN is associated with tumor cell proliferation in MES-GBM. Further research has revealed that the RAS-PI3K-AKT-p65 axis inside MES-GBM cells mediates the transcriptional activation of HOTAIR. HOTAIR, acting as a scaffold factor, recruits the PRC2 complex, leading to H3K27 trimethylation in the ERBIN promoter region, resulting in transcriptional repression of ERBIN.

When we overexpress ERBIN in MES-GBM cells, the abnormally activated RAS signaling is inhibited, leading to reduced levels of downstream cell cycle and EMT progression markers. These findings demonstrate the role of ERBIN as a tumor suppressor in MES-GBM. To restore ERBIN expression in MES-GBM, we focus on the interaction between HOTAIR and EZH2 as a research breakthrough that has been extensively studied 53, 54. In our previous study, ADQ and AQB were identified as small-molecule inhibitors capable of blocking the HOTAIR-EZH2 interaction, showing positive preclinical effects in GBM and other cancer models 41, 55, 56. EPIC-0412 was then designed and screened to further sensitize GBM cells to TMZ by blocking the HOTAIR-EZH2 interaction 32. In addition, our previous studies also found that EPE-0412 up-regulates ATF3 expression by inhibiting HOTAIR-EZH2 mediated H3K27 trimethylation, and perturbs the TCA cycle of GBM cells through the ATF3-SDHA axis 34.

Here, we found that EPIC-0412 can restore the epigenetic silencing effect of ERBIN by blocking the interaction between HOTAIR and EZH2, leading to increased expression of ERBIN in MES-GBM. This inhibits RAS signaling in MES-GBM cells and suppresses the proliferation and EMT progression of downstream cells. In all, our research provides an effective treatment for MES-GBM. By blocking the interaction between the HOATIR and PRC2 complex on chromatin, EPE-0412 produced different biological effects on GBM cells. In this study, the reversion of ERBIN silencing by EPIC-0412 is only one of all the effects, but all of EPIC's mechanisms of action are based on tumor epigenomics, which reveals the important role of epigenetic reprogramming in GBM cells in mediating cell tumor proliferation, chemotherapy resistance, and metabolic reprogramming. In conclusion, our study based on EPIC-0412 offers the possibility of targeting epigenome regulators to treat tumors.

RAS proteins are small GTPases cycling between the inactive GDP-bound to the active GTP-bound forms. This RAS-GTP/GDP cycling is positively regulated by the GTP exchange factors (GEFs), which facilitate the exchange of GDP to GTP, and are negatively regulated by the GTPase activating proteins (GAPs), such as NF1, promoting GTP hydrolysis to GDP. We identified RASGRP1 as a synthetically lethal gene of TMZ. Previous studies have reported that RASGRP1 plays an oncogenic role in several cancers, including leukemia, lymphoma, skin tumors, and colorectal cancer 45. We found that RASGRP1 activated the RAS signaling by promoting the RAS-GTP transformation, which then increased the downstream MYC protein expression and promoted expressions of DDR-associated genes in MES-GBM cells. Notably, we were able to achieve a significant TMZ sensitization effect when RASGRP1 was knocked down in GBM cells. In our study, RASGRP1 was the first reported oncogene related to GBM, which could serve as a target for the combination drug therapy.

MVBs are intracellular single membrane-bound organelles that play pivotal roles in a variety of biological processes, including protein transport, recycling, degradation, and extracellular vesicle secretion 57-59. The biogenesis of MVB is mediated by the endosomal sorting complex required for transport (ESCRT) complexes. There are four major subtypes of this complex system: ESCRT-0, ESCRT-I, ESCRT-II, and ESCRT-III 60. VPS28 is a component of the ESCRT-I complex that regulates MVB-dependent cargo sorting 60, 61. It has been shown that loss of VPS28 function reduces the endosomal transport of Awd in Drosophila larvae fat cells 62. Our previous studies have demonstrated that GBM cells can regulate the intracellular TMZ concentration through extracellular vesicle-mediated TMZ efflux 48. Here, we found that VPS28 mediated the Exos synthesis and secretion by promoting the early endosome formation and construction of MVBs, which subsequently reduced the intracellular therapeutic dose of TMZ and enhanced its efflux in the MES-GBM cells. VPS28 KD restored the intracellular therapeutic concentration of TMZ by inhibiting MVB processing as well as the TMZ-loaded Exos secretion in GBM cells. Previously few studies revealed the oncogenic role of VPS28 in tumors. Here, we found another novel function of VPS28 as a synthetic lethal gene for TMZ, providing a new target for overcoming the MES-GBM chemoresistance.

There are currently no small-molecule inhibitors against RASGRP1 and VPS28, so we developed a blood Exos-based delivery system to load siRNAs targeting these two genes. In our previous study, a drug, metformin, and a siRNA were loaded into blood-derived Exos and showed promising anti-tumor effects *in vivo*. Here, we explored the new delivery method for transporting TMZ and EPIC together across the BBB, and a delivery system containing two drugs and siRNAs was validated *in vivo*. This drug delivery system is likely to solve the problem of targeted siRNA delivery to tumors and rapid drug degradation, which overall can improve the therapeutic efficacy in GBM patients. This blood-derived exosome delivery system can also be customized to personalized treatments by loading specific drugs according to the patient's genetic background, which provides a novel approach to the treatment of GBM and can be extended to any cancer.

## Conclusions

**Figure 10:**
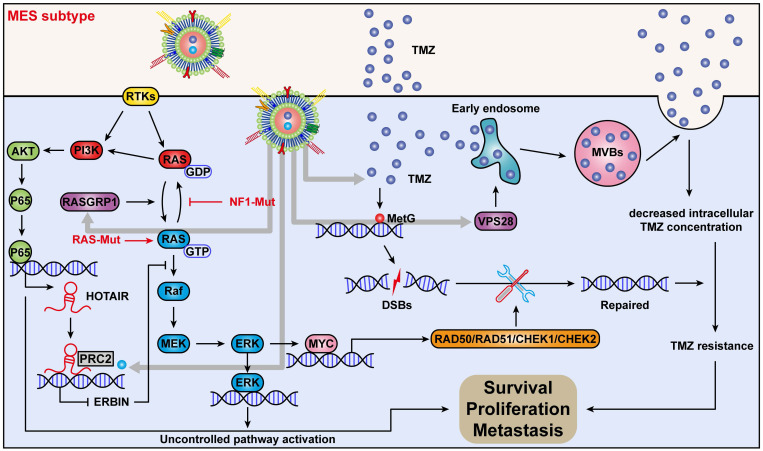
Mechanism of action of quadruple combination therapy based on the targeted Exos delivery system.

In conclusion, we discovered a new mechanism by which *ERBIN* expression could be epigenetically silenced by the RAS signaling in the MES-GBM subtype. Restoration of the ERBIN expression by EPIC-0412 significantly inhibited the downstream RAS signaling. Moreover, RASGRP1 and VPS28 were identified as the TMZ resistance genes, that could enhance the RAS-GDP to RAS-GTP transition and TMZ efflux. We constructed a quadruple combination therapy based on the targeted Exos delivery system that demonstrated a significant reduction in the tumor burden *in vivo* (**Figure 10**). Therefore, our study provides new insights and therapeutic approaches for tumor progression and TMZ resistance in the MES subtype of GBM.

## Supplementary Material

Supplementary figures and tables.
