# Supplementary material for: CRISPR-Cas9 library screening combined with an exosome-targeted delivery system addresses tumorigenesis/TMZ resistance in the mesenchymal subtype of glioblastoma
Source: Theranostics. 2024 Apr 29;14(7):2835–55. doi: 10.7150/thno.92703 (PMC11103500; doi:10.7150/thno.92703)
Supplement: Supplementary file 1 — Supplementary figures and tables. [file thnov14p2835s1.pdf]

Supplementary figures and tables.

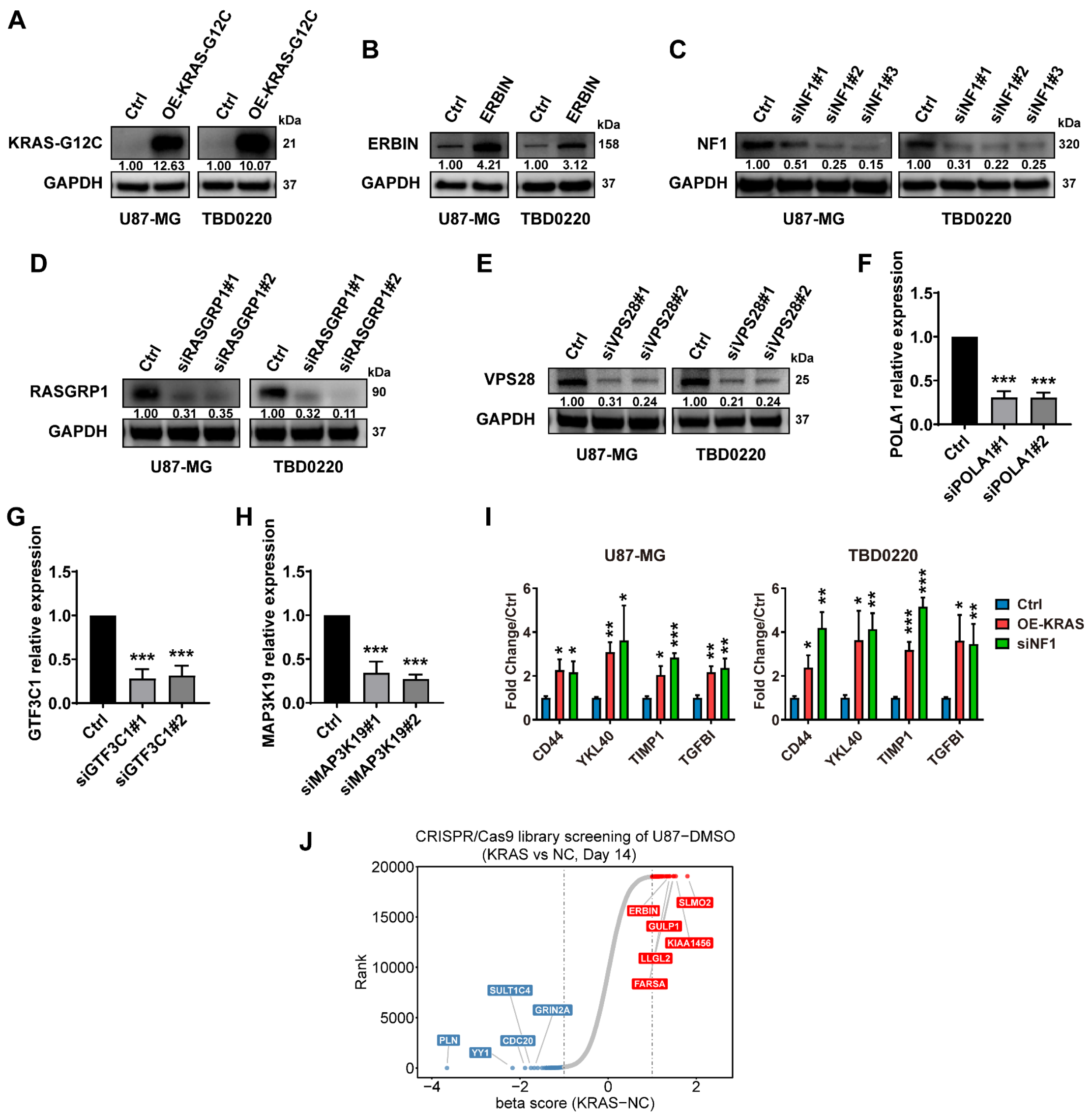

Figure S1. The WB analysis showed the overexpression efficiency of (A) KRAS-G12C (B)ERBIN in GBM cells. The WB analysis showed the knockdown efficiency of (C) NF1, (D) RASGRP1, and (E)VPS28 in GBM cells. The qRT-PCR analysis confirmed the knockdown efficiency of (F)POLA1, (G) GTF3C1, (H) MAP3K19 in GBM cells. (I) Relative mRNA levels of MES genes of OE-KRAS or siNF1 GBM cells. (J) Ranked plots of genes from CRISPR/Cas9 library screening of U87-DMSO cells. Data are represented as mean ± s.d.; n = 3 independent experiments. (***P < 0.001, **P < 0.01, *P < 0.05).

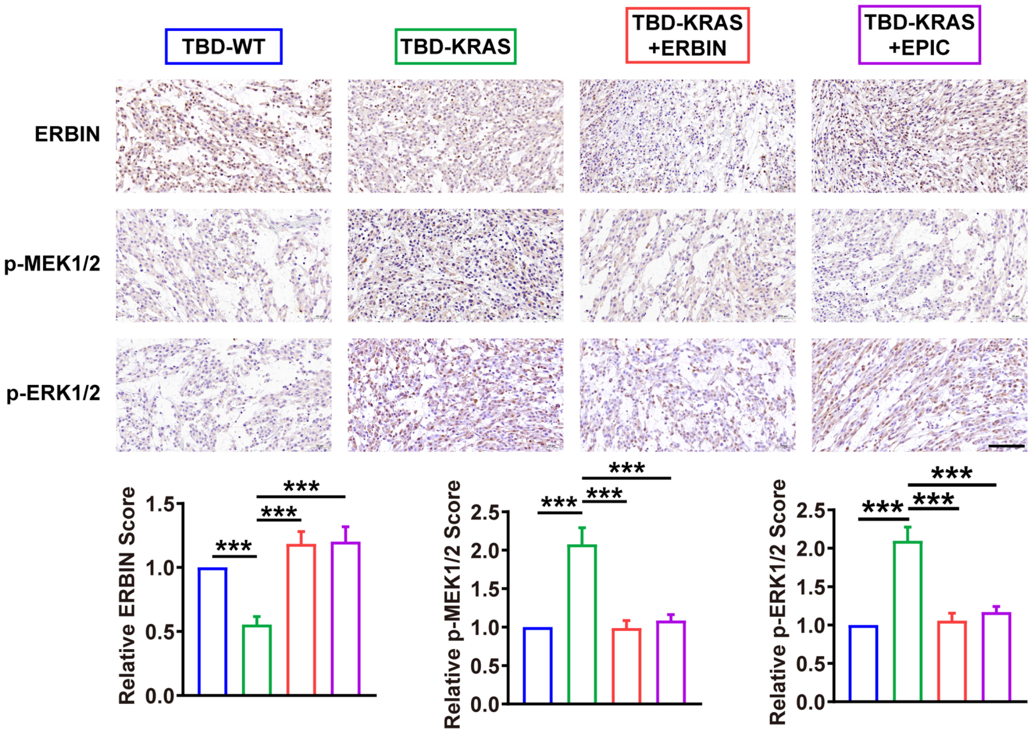

Figure S2. IHC staining of brain sections of tumor-bearing mice. Scale bar, 100 μm. Error bars indicate mean ± s.d.; ***P < 0.001.

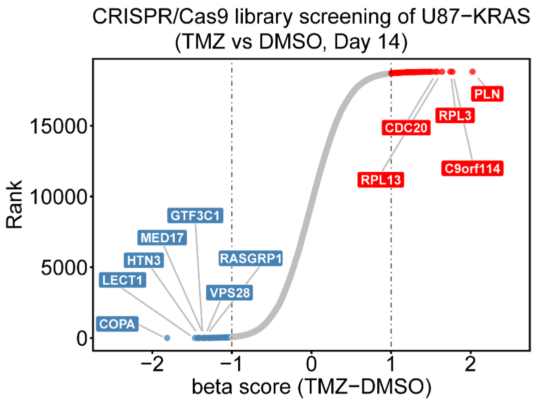

Figure S3. Ranked plots of genes from CRISPR/Cas9 library screening of U87-KRAS cells.

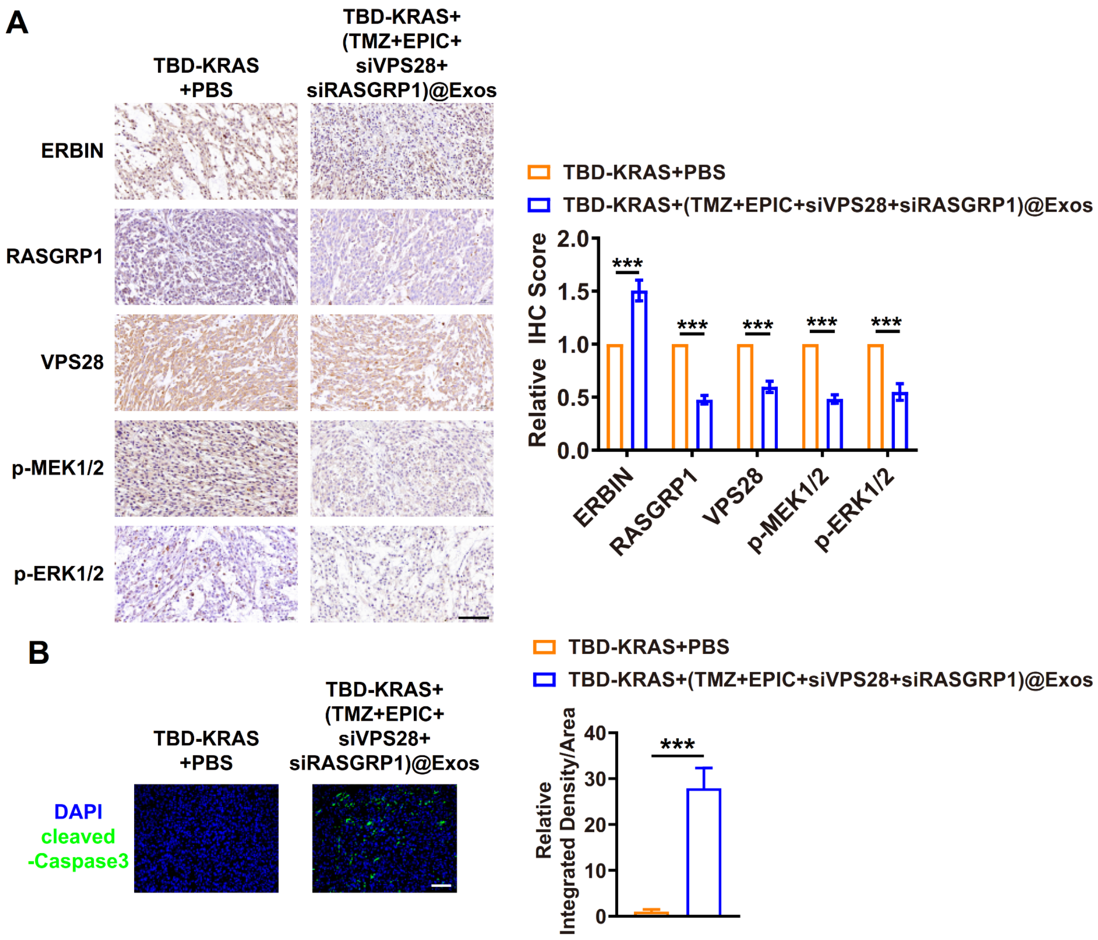

Figure S4. (A) IHC staining of brain sections of tumor-bearing mice. Scale bar, 100 μm. (B) IF staining of cleaved-Caspase 3 of brain sections from tumor-bearing mice. Scale bar, 100μm. Error bars indicate mean ± s.d.; ***P < 0.001.

Table S1.

Sequences information	
**siRNA oligos**	
siNF1#1-F	CAGUGAACGUAAGGGUUCUUU
siNF1#1-R	AGAACCCUUACGUUCACUGUU
siNF1#2-F	GGAAUAAGAUGGUAGAAUACC
siNF1#2-R	UAUUCUACCAUCUUAUUCCUA
siNF1#3-F	GAUAGAAGCUACAGUAAUAGC
siNF1#3-R	UAUUACUGUAGCUUCUAUCAG
siRASGRP1#1-F	GGGAUGAGAUCACAGCCUACU
siRASGRP1#1-R	UAGGCUGUGAUCUCAUCCCUG
siRASGRP1#2-F	AGAUUGCUGCGAGUUUUCCAUUU
siRASGRP1#2-R	AUGGAAAACUCGCAGCAAUCUUU
siVPS28#1-F	GGCUCCUGGUCCAAUACAAAG
siVPS28#1-R	UUGUAUUGGACCAGGAGCCGG
siVPS28#2-F	GAAGUGAAGUUGUACAAGAAC
siVPS28#2-R	UCUUGUACAACUUCACUUCCU
siPOLA1#1-F	GGAGAUACAGAUUCAAUUAUG
siPOLA1#1-R	UAAUUGAAUCUGUAUCUCCAU
siPOLA1#2-F	GCAUGAAGACAAUGCAGAAUG
siPOLA1#2-R	UUCUGCAUUGUCUUCAUGCUG
siGTF3C1#1-F	GCUUCUACGGACACCUCAAGC
siGTF3C1#1-R	UUGAGGUGUCCGUAGAAGCUG
siGTF3C1#2-F	CGGAGACAGUGUAUGUCGACG
siGTF3C1#2-R	UCGACAUACACUGUCUCCGUG
siMAP3K19#1-F	CAUUGGUUGUACUGUGUUUGA
siMAP3K19#1-R	AAACACAGUACAACCAAUGCU
siMAP3K19#2-F	GGAGUAAGUUGGAUUCAAAGA
siMAP3K19#2-R	UUUGAAUCCAACUUACUCCUA
**qRT-PCR Primers**	
HOTAIR-F	CCAGAGAACGCTGGAAAAACCTG
HOTAIR-R	GGAGATGATAAGAAGAGCAAGGAA
ERBIN-F	GTCAAGACACCTCACTCTGCTC
ERBIN-R	CATGAGCTTTGAACTTTTCCTCTG
RAD50-F	GGAAGAGCAGTTGTCCAGTTACG
RAD50-R	GAGTAAACTGCTGTGGCTCCAG
RAD51-F	TCTCTGGCAGTGATGTCCTGGA
RAD51-R	TAAAGGGCGGTGGCACTGTCTA
CHEK1-F	GTGTCAGAGTCTCCCAGTGGAT
CHEK1-R	GTTCTGGCTGAGAACTGGAGTAC
CHEK2-F	GACCAAGAACCTGAGGAGCCTA
CHEK2-R	GGATCAGATGACAGCAGGAGTTC

GTF3C1-F	GCTCGATTCCAGCTTCTACGGA
GTF3C1-R	ATGTCTGGAGCCTCACTGTGAG
POLA1-F	GGACCAACACATCTAGCCTGGA
POLA1-R	GGTCTGGTTTCAAAGCCATTGCC
MAP3K19-F	GCAACCCAGGACAGAAGAGTTT
MAP3K19-R	GACAGTGGTGGCAGAAGGAA
**ChIP-PCR Primers**	
HOTAIR-ChIP-F	GCTTTGGGATTTTCAGGTCA
HOTAIR-ChIP-R	AGAGGTGGAAGCCAGGAACT
ERBIN-ChIP-F	GAATGGGGGTGACGAATGGT
ERBIN-ChIP-R	GGCAGGGTTCGCGTTTATTG
RAD50-ChIP-F	ACAGGTCTTGGTCTCCCCTT
RAD50-ChIP-R	CTAGGTGCTGGGTGCTGTTG
RAD51-ChIP-F	CCCGTACGCTAGCTCCATTT
RAD51-ChIP-R	GAGCCTCCTACTCGCATTCC
CHEK1-ChIP-F	CTCCCAACACGGAGTTCCTC
CHEK1-ChIP-R	ACAGTCGGTGAAGCAGAGTG
CHEK2-ChIP-F	AAAGGAAGAGAGTCGCCCAC
CHEK2-ChIP-R	GTTGGGTGTTTGGTGGTTCG
**ChIRP-PCR Primers**	
ERBIN-ChIRP-F	GAATGGGGGTGACGAATGGT
ERBIN-ChIRP-R	GGCAGGGTTCGCGTTTATTG

Table S2.

Antibodies			
**Name**	**Company**	**Cat**	**Application**
EZH2	Cell Signaling Technology	5246	WB (1:1000)
GAPDH	Affinity Biosciences	AF7021	WB (1:1000)
ERBIN	abcam	ab247081	WB (1:1000), IHC (1:400)
AKT	Cell Signaling Technology	9272	WB (1:1000)
p-AKT (Ser473)	Cell Signaling Technology	4060	WB (1:1000)
p65	Cell Signaling Technology	8242	WB (1:1000), ChIP (1:100)
p-p65 (Ser536)	Cell Signaling Technology	3033	WB (1:1000)
c-Raf	Affinity Biosciences	AF6065	WB (1:1000)
p-c-Raf (Ser338)	Affinity Biosciences	AF3065	WB (1:1000)
MEK1/2	Cell Signaling Technology	4694	WB (1:1000)
p-MEK1/2 (Ser217/221)	Cell Signaling Technology	3958	WB (1:1000)
ERK1/2	Cell Signaling Technology	4695	WB (1:1000)
p-ERK1/2 (Thr202/Tyr204)	Cell Signaling Technology	4370	WB (1:1000), IHC (1:400)
Caspase3	Cell Signaling Technology	9662	WB (1:1000)
cleaved-Caspase3	Cell Signaling Technology	9664	WB (1:1000)
Caspase7	Cell Signaling Technology	9492	WB (1:1000)
cleaved-Caspase7	Cell Signaling Technology	8438	WB (1:1000)
E-Cadherin	Cell Signaling Technology	3195	WB (1:1000)
N-Cadherin	Cell Signaling Technology	13116	WB (1:1000)
Vimentin	Cell Signaling Technology	5741	WB (1:1000)
CDK4	Affinity Biosciences	DF6102	WB (1:1000)
CDK6	abcam	ab124821	WB (1:50000)
CyclinD1	Affinity Biosciences	AF0931	WB (1:1000)

Rb	Affinity Biosciences	DF6840	WB (1:1000)
p-Rb (Ser780)	Affinity Biosciences	AF3103	WB (1:1000)
KRAS-G12C	ABclonal	RP02973LQ	WB (1:1000)
MYC	Cell Signaling Technology	18583	WB (1:1000), ChIP (1:100)
Ki67	abcam	ab15580	IHC (1:1000)
γ-H2AX (phospho S139)	abcam	ab11174	WB (1:2000), IF (1:500), IHC (1:1000)
RAD50	Affinity Biosciences	DF7176	WB (1:1000)
RAD51	abcam	ab133534	WB (1:10000)
CHK1	Affinity Biosciences	AF6004	WB (1:1000)
CHK2	Affinity Biosciences	AF6033	WB (1:1000)
Alix	Cell Signaling Technology	92880	WB (1:1000)
Tsg101	Proteintech	28283-1-AP	WB (1:2000)
CD63	Proteintech	25682-1-AP	WB (1:1000)
CD9	Proteintech	20597-1-AP	WB (1:1000)
Calnexin	Proteintech	10427-2-AP	WB (1:5000)
CD81	Cell Signaling Technology	10037	WB (1:1000)
TfR	Affinity Biosciences	AF5343	WB (1:1000)
CD31	abcam	ab76533	IF (1:500)
EEA1	Cell Signaling Technology	48453	IF (1:200)
NF1	Cell Signaling Technology	14623	WB (1:1000)
RASGRP1	ABclonal	A10495	WB (1:1000)
VPS28	abcam	ab154793	WB (1:1000)
Phospho-MEK1/2 (Ser221)	Cell Signaling Technology	2338	IHC (1:50)
